# Modern Risk Stratification of Acute Myeloid Leukemia in 2023: Integrating Established and Emerging Prognostic Factors

**DOI:** 10.3390/cancers15133512

**Published:** 2023-07-06

**Authors:** Eleonora Boscaro, Irene Urbino, Federica Maria Catania, Giulia Arrigo, Carolina Secreto, Matteo Olivi, Stefano D’Ardia, Chiara Frairia, Valentina Giai, Roberto Freilone, Dario Ferrero, Ernesta Audisio, Marco Cerrano

**Affiliations:** 1 Division of Hematology, Department of Oncology, Presidio Molinette, AOU Città della Salute e della Scienza di Torino, 10126 Turin, Italy; eboscaro@cittadellasalute.to.it (E.B.); iurbino@cittadellasalute.to.it (I.U.); fcatania@cittadellasalute.to.it (F.M.C.); garrigo@cittadellasalute.to.it (G.A.); csecreto@cittadellasalute.to.it (C.S.); molivi@cittadellasalute.to.it (M.O.); sdardia@cittadellasalute.to.it (S.D.); cfrairia@cittadellasalute.to.it (C.F.); vgiai@cittadellasalute.to.it (V.G.); rofreilone@cittadellasalute.to.it (R.F.); eaudisio@cittadellasalute.to.it (E.A.); 2 Division of Hematology, Department of Molecular Biotechnology and Health Sciences, University of Torino, 10126 Turin, Italy; dferrero@cittadellasalute.to.it

**Keywords:** acute myeloid leukemia, prognosis, precision medicine, measurable residual disease, clonal architecture

## Abstract

**Simple Summary:**

Several factors, both patient- and disease-related, are essential to accurately estimate acute myeloid leukemia (AML) prognosis. The rapidly evolving field, from both the genetic and therapeutic standpoints, and the availability of measurable residual disease (MRD) data have made traditional prognostic factors less reliable. Consequently, updated recommendations are trying to recapitulate the current scenario, but several questions remain to be answered, including the impact of complex co-mutational patterns and the role of clonal architecture. In the present review, we summarize established and new AML risk factors, and we discuss the emerging comprehensive approaches to effectively integrate all relevant prognostic data to better inform patient care.

**Abstract:**

An accurate estimation of AML prognosis is complex since it depends on patient-related factors, AML manifestations at diagnosis, and disease genetics. Furthermore, the depth of response, evaluated using the level of MRD, has been established as a strong prognostic factor in several AML subgroups. In recent years, this rapidly evolving field has made the prognostic evaluation of AML more challenging. Traditional prognostic factors, established in cohorts of patients treated with standard intensive chemotherapy, are becoming less accurate as new effective therapies are emerging. The widespread availability of next-generation sequencing platforms has improved our knowledge of AML biology and, consequently, the recent ELN 2022 recommendations significantly expanded the role of new gene mutations. However, the impact of rare co-mutational patterns remains to be fully disclosed, and large international consortia such as the HARMONY project will hopefully be instrumental to this aim. Moreover, accumulating evidence suggests that clonal architecture plays a significant prognostic role. The integration of clinical, cytogenetic, and molecular factors is essential, but hierarchical methods are reaching their limit. Thus, innovative approaches are being extensively explored, including those based on “knowledge banks”. Indeed, more robust prognostic estimations can be obtained by matching each patient’s genomic and clinical data with the ones derived from very large cohorts, but further improvements are needed.

## 1. Introduction

**Figure 1:**
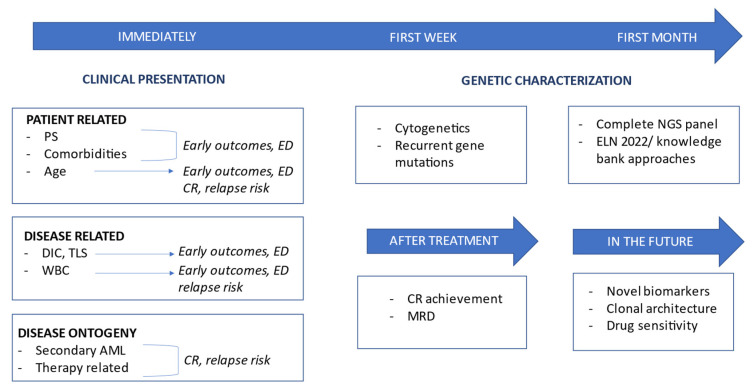
Graphical representation showing dynamic risk estimation of AML in 2023. Abbreviations: PS, performance status; ED, early death; CR, complete response; DIC, disseminated intravascular coagulation; TLS, tumor lysis syndrome; WBC, white blood cell; MRD, minimal residual disease; NGS, next-generation sequencing; ELN, European leukemia net.

The estimation of prognosis in acute myeloid leukemia (AML) is the result of a multilayered, integrated evaluation, which should consider several factors, both clinical, such as patient characteristics and disease manifestations at time of presentation, and biological ones (e.g., cytogenetic abnormalities and gene mutations). In addition to baseline variables evaluable at diagnosis, prognostic stratification of AML patients should consider evolutive parameters, such as measurable residual disease (MRD) at different pre-defined time points during therapy (Figure 1).

Several steps contribute to AML outcome, including early death rate, refractoriness to induction, disease relapse, response after salvage therapy, and treatment-related mortality, and distinct prognostic variables impact differently on them. Indeed, disease characteristics at diagnosis and patient clinical conditions strongly impact early mortality, while the AML genetic background could predict the likelihood of achieving remission and the risk of relapse. 

Importantly, the impact of each prognostic factor can be influenced by therapy, and several discrepancies among published studies could be due to differences in treatment intensity or the drugs used, both in induction and during post-remission therapy (e.g., chemotherapy only versus allogeneic hematopoietic cell transplantation, HCT). This concept has become even more relevant in recent years, since new agents have been added to standard intensive chemotherapy regimens (e.g., midostaurin) and novel formulations of drugs have been approved (e.g., CPX-351), which may modify the prognostic value of different clinical and biological factors. In addition, effective combinations of non-intensive treatments, such as hypomethylating agents (HMAs) plus venetoclax, are being increasingly used (mostly) for unfit patients, but assuming that traditional prognostic factors could play the same role in this new therapeutic context without adequate validation could be misleading. 

Treatment decisions are highly influenced by prognostic stratification, in particular, the allocation to HCT is usually limited to patients predicted to have a high risk of relapse with chemotherapy consolidation. However, it is important to consider that prognostic stratification in a specific therapeutic context significantly differs from a theragnostic-oriented approach. In the present review, we will focus on prognosis only, as an extensive discussion on how prognostic factors influence treatment choices can be found elsewhere [1,2].

Biological prognostication of AML has relied mostly on cytogenetics for a long time. In recent years, an increasing number of gene mutations have emerged as prognostic-relevant, initially in patients with normal karyotype and then regardless of cytogenetics, challenging conventional hierarchical risk stratification models. Furthermore, other factors such as gene expression signatures and clonal architecture are being confirmed as extremely relevant to further improve stratification accuracy. Finally, the role of a dynamic parameter such as MRD and its integration with baseline factors is being actively explored. Here, we review the established AML prognostic factors, and we discuss the emerging ones, focusing on innovative comprehensive approaches aiming to integrate and weight them. Summarizing the prognostic risk stratification of AML in specific contexts, such as children or relapsed patients, goes beyond the scope of this review, and these subjects have been recently reviewed elsewhere [3,4].

## 2. Clinical Risk Profile

### 2.1. Patient-Related Risk Factors: Age, Performance Status, and Frailty

Age has been confirmed as one of the most relevant determinants of AML outcome in virtually all published studies, with 5-year survival for patients above 60 years not exceeding 10–15% even in recent reports, and particularly dismal results for those above 70 years [5]. On the one hand, this consistency is due to the strong association between advanced age and adverse risk cytogenetic abnormalities and gene alterations, such as complex karyotype, myelodysplasia-related gene mutations, and secondary disease [6,7,8].

On the other hand, older patients often present with poor performance status and several comorbidities, which increase the chance of early death and treatment-related complications, also reducing the possibilities of effective salvage treatment in case of relapse or refractoriness to initial therapy [5,8].

Performance status is an easy and instantaneous picture of general conditions, with a clear impact on early mortality, the chance to achieve a complete response, and long-term survival [7]. Indeed, performance status is strongly correlated to age and comorbidities, but it could also be determined by disease presentation, and could sometimes significantly improve when initial complications are resolved and treatment is started [9].

Comorbidities are evaluated using different scores able to identify patients who would not benefit from intensive chemotherapy [10], which could also be used to estimate patients’ outcomes [1]. Indeed, simple parameters such as NT-proBNP could be particularly helpful for identifying patients at higher risk of early mortality [11]. Frailty is better evaluated using geriatric-inspired assessment tools, which could capture weaknesses sometimes difficult to fully understand using clinical evaluation only [12,13].

### 2.2. Disease-Related Risk Factors: Disease Presentation, Extramedullary Disease, and Inaugural Complications

In the context of AML, hyperleukocytosis (leucocytes >50,000 or 100,000/mL, according to different study groups) is present in 5–20% of cases, and it is associated with an elevated risk of complications and early death [14]. Indeed, severe inaugural complications such as leukostasis, tumor lysis syndrome, and disseminated intravascular coagulation could jeopardize the possibilities of successful treatment. These types of complications, which are strongly associated with hyperleukocytosis, in addition to severe infections, should be therefore carefully assessed, prevented when possible, and promptly treated [15,16,17]. Furthermore, even accounting for genetic risk factors, a higher leucocyte count seems to be associated with an increased relapse risk and inferior survival, although to a lesser extent than initially hypothesized [18,19,20].

Extramedullary disease, including central nervous system localizations, is associated with hyperleukocytosis, but its prognostic relevance is not firmly established. Indeed, the largest report published so far did not find an independent prognostic value after accounting for genetics and leukocyte count [21].

### 2.3. Disease Ontogeny

Considering prior disease history, two ontological families can be distinguished: de novo and secondary AML (sAML).

Secondary AML is itself a heterogeneous group, which includes AML deriving from an antecedent hematological disorder, namely myelodysplastic syndrome (MDS), myeloproliferative neoplasm (MPN), or aplastic anemia (AA), and therapy-related AML (t-AML), as a late adverse effect of prior cytotoxic chemotherapy or radiotherapy [22].

Inferior outcomes and lower response rates to intensive chemotherapy have been consistently reported [23,24], with a higher incidence of high-risk cytogenetics and a different genetic signature when compared to de novo AML [25]. Among t-AML, patients with previous treatment with topoisomerase-II inhibitors present a higher incidence of balanced translocations [26,27]. On the other hand, t-AML induced using alkylating agents is characterized by a high frequency of *TP53* and *PPMD1* mutations [28].

Although globally associated with inferior outcomes, response to treatment and prognosis can vary considerably among patients. Along with clinical differences (e.g., AML evolved from an MPN, and possibly AA [29], are generally associated with even worse outcomes compared to AML secondary to MDS [23]), the genetic profile plays a crucial role. Unfavorable cytogenetic subtypes are overrepresented in sAML, and cytogenetic risk classification remains a major determinant of the outcome for sAML patients. Indeed most, but not all [30], studies suggested that the prognostic impact of sAML could lose its significance when cytogenetic risk is considered [31]. Specifically, favorable rearrangements such as t(15;17) or core binding factor (CBF) translocations induced by topoisomerase-II inhibitors exposures retain their favorable prognosis also for sAML, although to a lesser extent for CBF AML [32,33,34,35]. Consistently, adverse risk mutations maintain their adverse impact in sAML [36], with a recent study showing that the prognostic impact of secondary versus de novo ontogeny is predominantly, albeit not completely, accounted for using the ELN 2022 risk classification [37].

Given the extreme relevance of gene mutations and the strong association with ontogeny [27], according to the recent International Consensus Classification (ICC), the presence of *ASXL1*, *BCOR*, *EZH2*, *RUNX1*, *SF3B1*, *SRSF2*, *STAG2*, *U2AF1*, or *ZRSR2* mutations defines the category of AML with myelodysplasia-related gene mutations and identifies a high-risk subgroup according to the ELN 2022 recommendations [38,39] (see below).

Finally, the group of treated sAML, i.e., occurring in patients who received an active treatment during the previous disease phase (e.g., hypomethylating agents for MDS), should also be recognized, since it is characterized by particularly poor outcomes [40].

## 3. Genetic Risk Profile

The advances in understanding the biological mechanisms behind AML oncogenesis have uncovered complex interactions between cytogenetic aberrations and gene mutations, which are essential for an accurate prognostic estimation. Thus, risk classifications have moved away from a hierarchical approach, in which gene mutations were only considered in cytogenetically normal AML, to a more integrated one.

### 3.1. Cytogenetics

Cytogenetic lesions and copy number alterations are reported in 50–60% of AML, and their role in determining risk stratification is a mainstay in biology-driven medicine [41], being confirmed in ELN and NCCN guidelines [39,42,43].

#### 3.1.1. Favorable Risk Recurrent Rearrangements

The best example of good cytogenetic risk leukemia is represented by acute promyelocytic leukemia (APL), identified with t(15;17) in the vast majority of cases [44]. Given the uniqueness of this entity, it will not be further discussed in this report.

The other recognized favorable cytogenetic risk group is represented by CBF leukemias, including AML with t(8;21)(q22;q22) and inv(16)(p13.1q22) or t(16;16)(p13.1;q22), whose fusion products are *RUNX1::RUNX1T1* and *CBFB::MYH11*, respectively [45,46]. CBF leukemias represent 10–15% of newly diagnosed AML and are characterized by younger age at diagnosis, de novo ontogeny, and a high probability of response after intensive chemotherapy, with a CR rate of 85–90% [47]. However, the group of CBF AML is heterogeneous in terms of additional cytogenetic abnormalities (ACAs) and associated gene mutations, with relevant differences between *RUNX1::RUNX1T1* and *CBFB::MYH11* AML [48,49,50]. Globally, while several reports suggested better long-term survival for *CBFB::MYH11* [20,51,52], this finding was not consistent in all studies [47,48,53,54].

In the pivotal Medical Research Council (MRC) cytogenetic study on 5876 young adults with AML, including 705 cases of CBF leukemias [41], no significant survival impact of ACAs on OS was observed among these patients, consistent with other reports, possibly retaining the positive role of trisomy 22 in *CBFB::MYH11* cases [20,55,56,57].

Among co-occurring gene mutations, *c-KIT* has been the most widely studied, and its negative impact in *RUNX1::RUNX1T1* AML emerged in several [58,59,60,61] but not all [62,63] reports. Thus, whether its presence should reclassify t(8;21) AML patients to a higher-risk group remains controversial.

*FLT3*-ITD mutations are present in 10–20% of CBF leukemias [56], and some reports suggest a negative prognostic impact [55,64], possibly restricted to high allelic ratio (AR) cases [56], or when patients with inv(16) and trisomy 22 are excluded [64]. Nonetheless, in other works, *FLT3*-ITD was of negligible clinical significance in this context [63,65].

#### 3.1.2. Intermediate and Adverse Risk Recurrent Rearrangements

*KMT2A* (*MLL*) rearrangements occur in roughly 5% of AML, in the context of balanced translocations involving 11q23 breakpoints with various possible partner loci [41].

Among *KMT2A*-rearranged AML, t(9;11)(p21.3;q23.3)/*KMT2A::MLLT3* are associated with a higher response rate to intensive chemotherapy compared to other translocations [66] and are thus included in the intermediate risk category when using the ELN 2022 classification, unlike the remaining high-risk *KMT2A*-rearranged subtypes [39].

Among adverse cytogenetic lesions, t(6;9)(p23.3;q34.1)/*DEK::NUP214* is frequently characterized by bone marrow dysplasia and additional cytogenetic abnormalities. The frequent (70–80% of the cases) co-occurring *FLT3*-ITD aberrations lack a confirmed prognostic significance in this already poor-risk group [67,68].

AML with inv(3;3)(q21.3q26.2) or t(3;3)(q21.3;q26.2) depicts a subset of particular poor-outcome with long-term survival chances extremely unlikely with conventional treatments [41,69,70]. Other 3q26 rearrangements, thus involving the *MECOM* (*EVI1*) gene as well, are also associated with dismal prognosis and were added to the 2022 ELN adverse risk group [39,71].

AML with t(9;22)(q34.1;q11.2)/*BCR*::*ABL1* is now recognized by the fifth WHO and the ICC classification [22,38], but it remains challenging to distinguish it from chronic myeloid leukemia blast crisis [72]. This entity remains classified in the adverse-risk group [39], although ACAs may play a central role in defining the outcome in these cases, and the impact of the addition of tyrosine kinase inhibitors to treatment has not been fully addressed [73,74,75].

Recently, the rare t(8;16)(p11.2;p13.3)/*KAT6A::CREBBP* rearrangement was better characterized, and its poor prognostic significance was confirmed [39,76].

#### 3.1.3. Aneuploidies

Among partial deletions and monosomies, those involving chromosomes 5, 7, and 17 are well-established poor prognostic factors [39,41,42]. Among these recurrent abnormalities, which are more common in older patients and frequently co-occurring, cases with isolated del(7q) are usually considered at intermediate risk [39,43], consistent with MDS data [77].

Complex karyotype (CK) is commonly defined by the presence of three or more cytogenetic unrelated chromosome abnormalities in the absence of other recurring class-defining genomic lesions and is invariably associated with poor prognosis [41,43,78,79]. However, this group of patients is quite heterogeneous. The number of alterations can matter, and generally, each additional aberration worsens the prognosis [20,41]. While the MRC group required four or more abnormalities to define a complex karyotype [41], Stölzel and colleagues showed that patients with ≥ four abnormalities have an adverse risk per se, while the outcome for patients with three abnormalities was dependent on the presence of abnormalities of strong influence, such as chromosome 5, 7 or 17 deletions [79], consistent with previous studies [80]. As a matter of fact, depending on the chromosomes involved, CK can be further stratified into typical CK, which harbors deletions/monosomies of chromosomes 5, 7, and 17 [81] and presents with a higher degree of cytogenetic complexity, and commonly *TP53* mutations, and atypical CK, which is associated with slightly better outcomes [82].

The importance of monosomies has been confirmed both within and outside CK, in particular in cases with a monosomal karyotype (MK), defined by Breems and colleagues as the presence of two or more monosomies or autosomal monosomy together with at least another karyotype abnormality [83]. Indeed, MK has been consistently associated with a dismal prognosis, with long-term survival rates often below 5% [83,84] and with the co-occurrence of CK and MK associated with an inferior OS compared to the sole CK.

Finally, chromosome trisomies appear to exert a different impact, being more often associated with intermediate risk [85], with isolated trisomy 4 recently suggested as favorable [86]. Hyperdiploid karyotype, usually referred to as AML with 49–65 chromosomes, represents a heterogeneous group that should be differentiated from CK [57,87]. Indeed, the ELN 2022 excludes hyperdiploid karyotypes with three or more trisomies (or polysomies) without structural abnormalities from the adverse CK group [39].

### 3.2. Gene Mutations

#### 3.2.1. FLT3

Mutations in FMS-like tyrosine kinase 3 (*FLT3*) occur in approximately 30% of AML and are frequently associated with normal karyotype, *NPM1* mutations (40%), and *DEK::NUP214*-AML (70%) [88]. More than two-thirds of the cases present with an internal tandem duplication (*FLT3*-ITD), while the remaining ones harbor point mutations in the tyrosine kinases domain (TKD), more frequently in the D835 residue.

*FLT3*-ITD has been consistently associated with poor prognosis and high relapse risk, which can vary according to its AR (higher being worse, see also below), size (longer being worse), and location of ITD insertion (TKD1 site being worse) [86,89,90,91].

Recently, in the genetic classification proposed by Tazi and colleagues, *FLT3*-ITD showed independent prognostic information in each genic class and could upgrade risk for all intermediate-risk patients to adverse-risk [57].

*FLT3*-TKD mutations do not exert an independent prognostic impact [39], albeit with some conflicting results [92,93], possibly depending on the context (i.e., *CBF*, *NPM1* vs. *KMT2A*-PTD-positive AML) [20,90,94].

#### 3.2.2. NPM1

Nuclophosmine 1 (*NPM1*) mutation is a class-defining genetic lesion, as consistently confirmed [22,38,57]. It frequently occurs in the context of normal karyotype, and it is accompanied by additional gene mutations in almost 70 % of cases [20,95,96].

As mentioned above, *NPM1* and *FLT3* mutations often co-occur [88], and *NPM1*-related good prognosis is mostly restricted to those cases not harboring *FLT3*-ITD [97].

In addition, the presence of *DNMT3A* mutations in patients co-harboring *NPM1* and *FLT3*-ITD could identify a subgroup with poor outcomes [20,98]. Indeed, clearly depicting the impact of co-mutations in *NPM1*-mutated AML has been particularly challenging, with recent data suggesting that the co-occurrence of MDS-related gene mutations might translate into inferior survival [99]. Probably, very large patient cohorts will be required to robustly address this issue, and this effort is actively being pursued in the context of the HARMONY project (see below).

Cytogenetic abnormalities can occur in 15% of *NPM1*-AML and, while globally, they did not show a significant impact [100], Angenendt et al. demonstrated that high-risk chromosomal abnormalities (3.4% of the cases) significantly and independently worsen prognosis, moving these cases into the adverse-risk category [101].

Rarely, *NPM1*-mutations occur in the context of therapy-related AML (t-*NPM1*), but their genetic signature and prognosis overlapped with that of de novo *NPM1*-mutated AML, outlining the genetic and prognostic diversity between t-*NPM1* and t-AML [102]. In accordance, going beyond the 2016 WHO classification [103], the current 2022 International Consensus Classification and the fifth WHO classification of myeloid neoplasms both classify *NPM1*-mutated AML as such, independently of the previous clinical history [22,38].

#### 3.2.3. CEBPA

CCAAT/enhancer binding protein α (*CEBPA*) mutations are found in roughly 10% of AML patients [104], and only biallelic-mutated cases seem to be associated with favorable outcomes [105,106,107]. However, two large studies recently demonstrated that in-frame mutations occurring in the bZIP domain of the *CEBPA* site were associated with good prognosis, irrespective of their occurrence as biallelic or monoallelic, prompting a modification of the current classifications and prognostic stratifications [39,108,109].

*GATA2* mutations are frequently found in *CEBPA*-AML and, although sometimes associated with better outcomes, further confirmation is needed. Conversely, the presence of *WT1* and *TET2* mutations have been associated with lower response rates and survival in some reports [110,111], but their independent relevance is not firmly established [108].

#### 3.2.4. TP53

Mutations in this onco-suppressor gene are among the commonest in cancer and account for about 10% of AML cases. *TP53* mutations frequently co-occur with CK/MK and in therapy-related settings, showing invariably poor response to intensive chemotherapy and dismal outcomes, predicating even worse survival in the context of CK [20,36,112].

As boundaries between AML and MDS fade in current classifications [38], recent data indicate that *TP53*-mutated AML and MDS with excess blasts share similar characteristics and prognoses, suggesting they should be regarded as a specific molecular disease entity [112,113,114].

In MDS, multi-hit *TP53* disruption (in the subsets of multiple mutations, mutation plus 17p deletion, or mutation plus loss of heterozygosity) frequently presents with a higher rate of additional chromosomal abnormalities compared to monoallelic/single-hit mutation and significantly worse prognosis [115]. Indeed, the detrimental impact of multi-hit *TP53* was also shown in AML [112,116,117], in accordance with data on the negative impact of high *TP53* variant allele frequency (VAF) [118]. However, this finding was not confirmed in the large analysis by Tazi and colleagues [57], suggesting further research on this issue is needed.

#### 3.2.5. RUNX1, ASXL1, and Other Myelodysplasia-Related Gene Mutations

*RUNX1* mutations have been associated with reduced response and survival in several studies [119,120,121,122], with some contrasting results [20]. Similarly, *ASXL1* aberrations consistently showed an adverse prognostic impact [20,123,124], mainly when co-occurring with mutated *RUNX1* and other epigenetic modifiers. Thus, both gene mutations were introduced as poor prognostic markers in the ELN 2017 risk classification [125].

More recently, these genes together with *BCOR*, *EZH2*, *SF3B1*, *SRSF2*, *STAG2*, *U2AF1*, and *ZRSR2* were recognized in the ICC as determinants for the entity of “AML with myelodysplasia-related gene mutations” irrespective of previous disease history [38], and the prognostic relevance of this entity was recognized in the ELN 2022 recommendations [27,39]. However, patients harboring these sAML mutations seem to experience rather heterogeneous outcomes. The study by Tazi et al. suggested that among these cases (which included the aforementioned mutations plus *SETBP1* and *KMT2A*-PTD aberrations), the association with adverse prognosis was specific to patients with ≥two mutations (5-year survival rate of 16%, identified as sAML2,) while the minority of patients with a single gene mutation experienced an intermediate outcome (5-year survival rate of 37%) [57]. Furthermore, the availability of new treatments, including CPX-351, could further complicate the scenario, as this agent seems to work particularly well in patients harboring myelodysplasia-related gene mutations, potentially overcoming their adverse prognostic value [126,127].

#### 3.2.6. Other Genes

The role of several gene mutations has been explored in the last decade, mostly with inconclusive results [3,128,129].

*DNMT3A* mutations, strongly associated with age-related clonal hematopoiesis, were associated with unfavorable outcomes in older studies [130,131], but their role was not always confirmed, as their prognostic impact could be influenced by age, co-occurring molecular aberrations, and the type of mutations (i.e., R882 versus others) [20,122,132]. Recently, *DNMT3A* mutations were shown to be useful to refine the 2017 ELN stratification, since their presence worsen the prognosis in each subgroup [52].

Roughly 5% of AML cases harbor partial tandem duplication of *KMT2A* (*KMT2A*-PTD), which was associated with unfavorable prognosis in several studies [104,133]. However, this abnormality is not commonly recognized as an independent prognostic marker [39], possibly because of the weight of co-mutations [20] and some discordant results [122,134]. As mentioned above, this mutation was recently included in the s-AML group by Tazi and colleagues [57].

The *WT1* prognostic role has been suggested in some studies, but the inconsistency among reports prevented its uniform acceptance [20,135,136]. In the study by Tazi and colleagues, *WT1* mutations in the absence of other classifying events identified a specific AML cluster with intermediate risk [57,137].

Mutations of *PTPN11*, a regulator involved in RAS signaling, have been associated with poor prognosis in three recent reports [138,139,140]. However, these findings should be interpreted with caution, as the negative impact of *PTPN11* appeared restricted to *NPM1*-wild-type patients in one report [140] and to ELN2017 favorable cases in another one [139].

The *CREBBP* gene is rarely disrupted in AML and mainly rearranged with different translocation partners, most frequently *KAT6A* at 8p11 [76,141], see above. However, data about gene mutations, including single nucleotide variants, are less robust. Recently, the Children Oncology Group explored the role of these genetic lesions in a large retrospective cohort of pediatric and young adult de novo AML patients (aged 0–29.8 years), showing that *CREBBP*-disrupted cases experience worse event-free survival and increased relapse risk compared to wild type ones [142]. However, this preliminary finding needs confirmation in the adult population.

Among germline mutations predisposing to MDS and AML, those affecting *DDX41* are the most common ones and are often found in advanced age. Recently, *DDX41* mutations have been associated with increased complete remission rates and rather favorable survival, both in real-life analyses and prospective clinical trials [143,144].

## 4. Measurable Residual Disease

Although it is widely agreed that residual leukemic cells lead to recurrent disease in acute leukemias, the recognition of the role of MRD in AML has been slower compared to acute lymphoblastic leukemia (ALL), a disease in which MRD has been accepted as the strongest prognostic factor [145]. MRD assessment and clinical application are challenging in AML, in part because of its genetic and immunophenotypic heterogeneity. Thus, different MRD detection methods have been developed, namely multiparameter flow cytometry (MFC), polymerase chain reaction (PCR), and next-generation sequencing (NGS). In addition, it is known that not all AML mutations have clinical utility for MRD monitoring, such as those found in age-related clonal hematopoiesis (e.g., *DNMT3A*, *TET2*, *ASXL1*) or in germline predisposition syndromes (e.g., *DDX41*, *RUNX1*, *GATA2*). Moreover, mutations in signaling pathway genes (e.g., *FLT3*, *KIT*, *RAS*) likely represent residual leukemia when detected, but being often sub-clonal, they have a low negative predictive value. Finally, most studies exploring the prognostic value of MRD in AML are heterogeneous in terms of the patient population (age and AML subtypes), the timing of MRD assessment, and the source (peripheral blood vs. bone marrow) [146]. However, in 2017, the ELN introduced MRD response as a subcategory of CR, acknowledging that patients achieving MRD-negative CR after intensive chemotherapy experience better outcomes compared to MRD-positive ones [125], and in 2018, the first ELN MRD consensus guidelines addressed comprehensively the role of MRD in AML [3,147]. Indeed, a large 2020 meta-analysis including 81 publications with 11151 AML patients treated with intensive chemotherapy clearly demonstrated the strength of the association between MRD and survival outcomes, regardless of patient- and disease-related factors and methodologic variables. In this meta-analysis, the 5-year estimated OS for the MRD-negative group was 68% compared with 34% for the MRD-positive group. MRD negativity was associated with improvement in long-term survival outcomes in all evaluated subgroups across different clinical contexts [118]. Given the enormous interest in this field and the new evidence, in 2021, the ELN MRD consensus guidelines were updated, allowing hematologists to standardize the use of MRD testing in clinical practice and indicating the directions for future improvements. Recommendations were given on the MRD detection method to use in different AML subtypes, on the timing of MRD testing, and on the source to use. Moreover, the role of NGS in MRD assessment was more comprehensively covered [148] (Table 1). Finally, the MRD consensus recommendations were integrated into the recently published 2022 ELN AML guidelines for AML diagnosis and management [39].

### 4.1. MRD in Less-Intensively Treated Patients

While the prognostic role of MRD is established in young and fit patients receiving intensive chemotherapy, there is limited evidence for its clinical significance in patients treated with low-intensity prolonged regimens, probably reflecting the low chances of deep and prolonged remissions. Recently, the introduction of venetoclax-based combinations led to increased CR rates and response durations, prompting researchers to evaluate MRD impact in this setting as well [149].

In the VIALE-A study, MRD-negative responses were achieved in 41% (67/164) of patients obtaining composite CR, with significantly prolonged remission duration and survival compared to MRD-positive cases. Multivariate analysis confirmed that MRD-negative CR was a strong independent predictor of OS [150]. The role of MRD was also evaluated in patients treated with 10-day decitabine plus venetoclax with similar results, supporting the relevance of MRD evaluation in patients treated with HMA and venetoclax [151].

### 4.2. MRD and HCT

Several studies have shown that patients undergoing HCT in MRD-positive CR have worse survival and increased relapse risk compared to MRD-negative ones [152,153]. Buckley et al. addressed this issue in a meta-analysis including 19 transplant studies, confirming a robust association between pre-HCT MRD positivity and post [154]. Nonetheless, the 2021 MRD consensus guidelines recommend that pre-transplant MRD positivity should not be considered as a contraindication to HCT but, when feasible, myeloablative conditioning should be used [148].

## 5. Current Risk Stratification Algorithms

Risk stratification algorithms are widely used to estimate AML patients’ prognoses and make therapeutic decisions. Indeed, these simplified systems are built to summarize the available evidence and, importantly, to identify prognostic factors whose role is robust and reproducible enough to inform clinical practice. The classification proposed by ELN is probably the most widely used, including by the last version of the NCCN guidelines, and it has recently been updated [39,125,155]. The main differences between the 2022 and the 2017 classification systems are outlined in Table 2.

Probably the most relevant changes regarded *FLT3*-ITD AR and its interaction with *NPM1*. Patients harboring *FLT3*-ITD with low AR (i.e., 0.5 or less in the ratio of the area under the curve “*FLT3*-ITD” divided by the area under the curve “*FLT3*-wild-type”) with *NPM1* mutations and those without *NPM1* aberrations but with *FLT3*-ITD with high AR were categorized as favorable and adverse-risk using the 2017 classification, respectively [125]. Conversely, the 2022 recommendations classified all *FLT3*-ITD patients lacking favorable cytogenetics or adverse genetic lesions in the intermediate risk group, given the challenges posed by the standardization of the assay measuring AR, the evidence of a beneficial impact of midostaurin-based regimens in *FLT3*-ITD AML irrespective of AR and *NPM1* mutational status, and the increased role of MRD [39,156].

Furthermore, the ELN 2022 considered several of the studies discussed above to update its classification:

- Patients with in-frame bZIP *CEBPA* mutations are now considered favorable-risk irrespective of *CEBPA* biallelic or monoallelic mutational status.
- *NPM1*-mutated patients with adverse cytogenetics are considered at adverse risk.
- Hyperdiploid karyotypes with three or more trisomies without structural abnormalities are excluded from the group of CK.
- In addition to *RUNX1* and *ASXL1*, other MDS-related gene mutations (*BCOR*, *EZH2*, *SF3B1*, *SRSF2*, *STAG2*, *U2AF1*, and *ZRSR2*) are added as poor-risk prognostic markers in the absence of favorable risk genetics.
- New high-risk rearrangements are included, namely t(3q26.2;v)/*MECOM* and t(8;16)(p11;p13)/*KAT6A::CREBB*.
- At least a 10% VAF is required to classify patients as *TP53*-mutated.

Recently, the updated classification was validated in large cohorts of intensively treated younger de novo AML patients [137,157], although some refinements were proposed [158,159,160], suggesting further improvements are likely possible.

Conversely, a recent analysis of the VIALE-A outlined how the ELN classification does not adequately stratify patients treated with HMA and venetoclax, confirming that different prognostic algorithms will be needed in this therapeutic context. Thus, the author proposed a stratification system based on the mutational status of *TP53*, *N*/*K RAS,* and *FLT3*-ITD, which requires validation [161].

Finally, dynamic parameters, i.e., treatment response including MRD, are increasingly stressed in the guidelines (see also above), as they significantly modify baseline prognostic stratification, in addition to informing treatment decisions [39].

## 6. Emerging Biological Risk Factors

The prognostic impact of several biological factors has been explored in AML, although they have not entered clinical practice yet.

### 6.1. RNA

Long noncoding RNAs (lncRNAs), microRNAs (miRNAs), and circular RNAs (circRNAs) are three noncoding RNA molecules that regulate DNA transcription and translation [162]. The expression level of lncRNAs could predict AML outcome, with several lncRNAs associated with prognosis in relatively small studies [163,164]. Interestingly, a four-gene lncRNA expression signature was shown to predict outcome in AML independently of ELN risk stratification in a rather large study including a validation cohort [165] and, more recently, the prognostic role of a 37 lncRNA signature was demonstrated on over 1000 patients, mostly in the pediatric setting [166].

MiRNAs are involved in tumorigenesis both as oncogenes and tumor suppressors. In rather old studies, the up-regulation of miR-181a was associated with a favorable prognosis, while higher expression of miR-155, miR-196b, and miR-644 was associated with a shorter OS [167,168,169].

Finally, circular RNAs (circRNAs) can be overexpressed in AML, but data are scantier. CircPVT1 was shown to be overexpressed in AML harboring oncogene *MYC* amplification, but survival implications are yet to be proven [170].

### 6.2. Methylation

DNA hypermethylation and the subsequent inactivation of tumor suppressor genes play a key role in AML pathogenesis, with methylation genes (i.e., *DNMT3A*, *TET2*, *IDH1/2*) being among the most frequently mutated in AML [88].

Different cytogenetic subgroups of AML have distinct DNA methylation profiles [171], and DNA methylation signatures could sub-stratify large genetic groups, such as *NPM1*-mutated AML, possibly identifying new prognostically relevant disease entities [171,172]. Several studies have explored the clinical and prognostic implications of DNA methylation patterns, concluding that aberrant DNA methylation is independently associated with clinical outcomes. Indeed, patients with a higher proportion of methylation changes at diagnosis showed shorter time to relapse [173,174,175]. Finally, Luskin and colleagues developed a microsphere-based assay to assess DNA methylation status, generating a methylation-based risk score (M-score) that was independently associated with CR and OS in different AML cohorts [176,177].

### 6.3. Leukemia-Stem Cells

The persistence of leukemia stem cells (LSCs) plays a pivotal role in driving AML relapse; thus, assessing AML LSC gene expression signatures has been proposed as a method to further refine prognosis. Indeed, several signatures and scores have been proposed [178,179,180]. Among them, LSC17, a 17-gene stemness score, was associated with poor clinical outcomes in multiple AML cohorts, even in the context of ELN 2017 classification [181,182,183,184].

### 6.4. Proteomics

The impact of protein expression in AML has long been studied, with an early focus on proteins involved in chemotherapy resistance, such as P-glycoprotein (the *MDR1* gene product), whose hyperexpression was mostly associated with worse prognosis [185,186]. The prognostic impact of the expression of anti- (e.g., BCL-2) or pro- (e.g., BAX or BAX/BCL2 ratio) apoptotic proteins was suggested, despite some inconsistencies [187,188,189,190]. In addition, subsequent studies indicated that specific functional proteomic profiles were associated with outcomes [191].

Recently, Jayavelu and colleagues performed a large proteogenomic analysis on uniformly treated AML patients that included, in addition to in-depth quantitative proteomics, cytogenetic profiling and DNA/RNA sequencing. The authors identified five distinct proteomic AML subtypes, reflecting specific biological characteristics, which could not be recapitulated with genetics. Importantly, one subtype captured only in the proteome (Mito-AML) was characterized by high expression of mitochondrial proteins and was associated with poor prognosis, with low CR rates and shortened survival after intensive chemotherapy. Finally, functional analyses suggested that Mito-AML could be more responsive to venetoclax-based treatments [192].

### 6.5. BH3 Profiling

BH3 profiling is a functional approach that can predict the cellular dependence on anti-apoptotic proteins like BCL-2 or MCL-1, based on mitochondria depolarization in response to a panel of BH3 sensitizer peptides. BH3 profiling was hypothesized to predict response to chemotherapy in AML some years ago [193], and recently, Dal Bello et al. showed in a uniformly intensively treated cohort of older AML patients that mitochondrial blast priming predicted prolonged OS in non-adverse risk AML [194].

## 7. Prognostic Impact of Clonal Architecture in AMLs

Beyond the prognostic impact of individual mutations in AML, their association with a patient could refine prognosis prediction [195]. Allelic mutational status is important for some of them such as *TP53*, as discussed above. Specific combinations seem to be synergistic, such as *DNMT3A* and *IDH1/2*, as they frequently co-occur in AML [20], are associated with clonal dominance when they co-occur in single cells [196], and convey poorer prognosis compared to single-mutated cases [20,197]. Higher numbers of leukemic clones, determined using conventional cytogenetic analyses [198] or inferred from bulk sequencing with the number of driver mutations and the number of epialleles [199], have also been associated with shorter survival. Phylogeny structure and clone size distribution are also important. Clonal dominance, i.e., the preponderance of one clone over the others, correlates with poorer prognosis [195,200]. Branching architecture owing to the parallel evolution of signaling mutations (i.e., clonal interference) predicts higher relapse rates in core-biding factor AML [63]. Most of these correlations were performed using imperfect clonal architecture inference on bulk sequencing data. Recent technological developments now give access to information on single-cell mutation co-occurrence, deciphering the precise clonal composition of leukemic samples [196,201]. Single-cell DNA sequencing in a large cohort of AML patients will probably refine the prognostic impact of clonal architectures. However, these associations with prognosis are correlative, and it is likely that in some cases, they result from an underlying biological process rather than a direct role of clonal structure on treatment resistance [202]. Emerging multi-omic, single-cell protocols would probably shed some light on these complex mechanisms by linking clonal architecture and functional diversity of leukemic cells [203,204].

## 8. Global Risk Assessment in AML

Despite the effectiveness of current prognostic stratification algorithms, such as the ELN2022 one, other clinical parameters, such as age, leukocytosis, or performance status exert a relevant prognostic impact, as previously discussed. Indeed, they interact with genetic lesions and can influence patients’ outcomes [20]. In the last years, some recommendations for transplant in first CR have included several of these parameters together with genetic risk, weighting them against the risk of non-relapse mortality, thus proposing a form of integrated approach [205].

Another strategy to integrate cytogenetic, molecular, and clinical factors has been the development of scoring systems [206], which, however, could not keep up with the rapidly evolving molecular landscape, and whose use is not common in clinical practice.

Clearly, hierarchical step-by-step integration approaches including cytogenetic and molecular aberrations are no longer able to recapitulate the full spectrum of AML. First, not all mutations in a given gene exert the same impact, as clearly established for *FLT3* (ITD vs. TKD), but this may also be the case for *DNMT3A* (R882 vs. others) [207] or *KIT* (exon 8 vs. 17) [55]. Second, three (or more) gene interactions have been confirmed to be relevant for patients’ stratification [20,208]. Third, the reciprocal relation of mutations and their repartitions into clones seems to exert a meaningful prognostic impact (see above).

Thus, in recent years, machine learning approaches have been proposed to overcome these issues.

In the context of the European Union-funded HARMONY project, Hernández Sánchez and colleagues analyzed 1093 intensively treated *NPM1*-mutated patients, applying a machine learning algorithm developed to identify combinations of up to four co-mutated genes with a potential impact on OS. Using a heuristic search algorithm and bootstrap sampling, they estimated the impact of all possible gene combinations on OS. Combining the mutational status of a few genes, namely *TP53*, *FLT3-ITD*, *IDH*, *DNMT3A*, *PTPN11*, *N*/*K RAS*, and *RAD21*, the authors stratified *NPM1*-mutated cases into four groups with significantly different outcomes, thus proposing a new genetic stratification model for these patients [209].

A different emerging approach, aiming to integrate the most available prognostic information layers without relying on studies on specific genetic interactions, was first reported by Gerstung and colleagues. The authors developed a multistage model based on matched clinical and genomic data from more than 1500 AML patients [20], which could predict for each patient the probability of different causes of mortality, namely, death without remission, death without relapse, and death after relapse. Indeed, this “knowledge bank approach” (KB) was able to improve the prediction of patients’ outcomes compared to standard risk stratification systems [210]. Furthermore, this method could estimate the impact of HCT on these probabilities, and it was calculated that following this tailored approach, the same survival could be maintained by reducing the number of HCTs by 20–25%. The authors developed an online tool, which allows an accurate prognostic prediction even in cases of missing data (https://cancer.sanger.ac.uk/aml-multistage, accessed on 1 June 2023). Huet and colleagues were later able to validate the KB approach in a real-life setting, confirming that it outperformed the survival prediction achieved with current risk classifications and the robustness of the algorithm to missing data [211]. More recently, the Cancer and Leukemia Group B validated this approach using patients treated in their trials, suggesting that the integration of additional genetic factors such as atypical complex karyotype, infrequent recurrent balanced chromosome rearrangements, and mutational status of new genes could improve the performance of KB algorithms [212]. The Papaemmanuil lab recently updated a somehow similar patient-tailored clinical decision tool based on an even larger patient cohort, which relied on a smaller gene panel and on clinical features [57]. An online tool is also available (https://www.aml-risk-model.com/calculator, accessed on 1 June 2023).

Focusing on its ability to guide HCT decisions in first CR1, Fenwarth and colleagues analyzed the performance of the KB approach for intensively treated younger patients in the context of the ALFA-0702 clinical trial. Not only were the authors able to demonstrate the superiority of this method to standard prognostic stratifications, but a personalized and appropriate HCT decision was derived using the integration of the KB approach with ELN 2017 risk score and, importantly, *NPM1* MRD. Finally, the authors devised an online decision tool (https://alfa-group.shinyapps.io/alfa-hsct/ accessed on 1 June 2023) [213]. A French group was also able to validate the KB approach in elderly patients treated with intensive chemotherapy, although, in this context, it was not superior to their recently developed and validated ALFA decision tool [214,215]. Indeed, by combining cytogenetics and seven gene mutations, the authors were able to identify a “no-go” group of patients with dismal outcomes when treated with intensive chemotherapy (2-year OS of 3%), clearly distinct from the intermediate and the favorable-risk ones.

KB-based approaches have been shown to improve tailored therapeutic decisions; however, several limitations still exist. Since new effective treatments are being approved [216], the survival estimation using data on patients treated with “7 + 3”-like traditional chemotherapy programs might become inaccurate, as recently suggested by a real-life study [217].

Furthermore, inclusive cohorts are required, to avoid overlooking certain subgroups (e.g., elderly patients less often enrolled in clinical trials). Importantly, when focusing on a particular setting such as HCT, specific factors should ideally be considered, such as donor type and conditioning regimens [218].

Despite its enormous prognostic relevance, a detailed genetic characterization and its integration with clinical data cannot fully depict AML behavior, as all the aforementioned methods are far from reaching perfect accuracy. Indeed, functional assays could complement genetic risk stratification to identify patients achieving long-term survival with intensive therapy. With this approach, the direct exposure of patients’ AML primary cells at diagnosis to several drugs can undercover specific vulnerabilities and resistance patterns, which could be used to personalize therapeutic choices [219]. In their seminal study, Tayner and colleagues explored the correlation between drug sensitivity, mutational status, and gene expression signatures, suggesting the role of specific gene networks in determining drug response [220]. More recently, Dal Bello and colleagues developed a niche-like drug sensitivity screening assay combining physiologic hypoxia and mesenchymal stromal cell co-culture to overcome the limitations of standard cultures and to represent more closely the conditions in which drugs act in vivo. In addition to predicting the response to anthracycline–cytarabine induction chemotherapy in a cohort of *NPM1*-mutated AML patients, higher relative drug activity was associated with an independent positive impact on event-free survival in that cohort [221]. Although promising, drug screening approaches are currently restricted to specialized labs, and a prospective investigation in larger cohorts is required to confirm the role of functional precision oncology in AML.

## 9. Conclusions

Huge advances in AML biology have led to an increasing complexity in prognostic estimation, as newly discovered factors are entering an already challenging scenario. Furthermore, several new treatments have been approved in the last years, and the impact of prognostic factors established in cohorts of conventionally treated patients should be confirmed in the new therapeutic context, both with intensive and non-intensive therapies.

Thus, new machine learning-based tools are being developed to integrate established prognostic factors in an evolving therapeutic scenario of a relatively rare disease, hopefully increasing the prediction accuracy of current models, which remain limited at the single-patient level [222], to whom our efforts should be addressed.

## Figures and Tables

**Table 1:** Current MRD recommendations according to 2021 ELN consensus guidelines.

**Detection Methods**	
qPCR	*NPM1*-mutated AML, CBF AML (*RUNX1::RUNX1T1* or *CBFB::MYH11*)
MFC *	AML lacking a molecular marker
NGS	At present, there are insufficient data to recommend it as a stand-alone technique
**Timing Assessment**	
qPCR-MRD	In PB, after two cycles of chemotherapy; in BM, at the end of consolidation; and in BM, every 3 months; or in PB, every 4–6 weeks for 24 months after the end of consolidation
MFC-MRD	In BM, after two cycles of chemotherapy, at the end of consolidation and prior to HCT
**MRD-Driven Treatment Decisions**	
Additional consolidation strategies	(1) MRD-pos using MFC after two cycles of intensive chemotherapy or consolidation chemotherapy and prior to or after HCT; (2) MRD-pos with ≥2% *NPM1* mutant copies per *ABL1* copies measured in BM or transcript levels of *NPM1* or CBF fusions failed to reach a 3–4 log reduction in the same tissue after completion of consolidation chemotherapy; (3) MRD relapse.
No change in treatment	Patients with *NPM1*-mutated or CBF AML who have stable molecular MRD detection at low level (MRD-LL)

Abbreviations: qPCR: quantitative polymerase chain reaction, MFC: multiparameter flow cytometry, NGS: next-generation sequencing, AML: acute myeloid leukemia, CBF: core-binding factor, MRD: measurable residual disease, PB: peripheral blood, BM: bone marrow, HCT: allogeneic hematopoietic cell transplantation. * Combining the two available approaches: leukemia-associated immunophenotypes (LAIP) identification and difference from normal (DfN) strategy.

**Table 2:** ELN 2017 versus ELN 2022.

ELN 2017	ELN 2022	Comments
**Favorable Risk**		
t(8;21)(q22;q22.1)/*RUNX1::RUNX1T1*	t(8;21)(q22;q22.1)/*RUNX1::RUNX1T1*	
inv(16)(p13.1q22) or t(16;16)(p13.1;q22)/*CBFB::MYH11*	inv(16)(p13.1q22) or t(16;16)(p13.1;q22)/*CBFB::MYH11*	
Mutated *NPM1* without *FLT3*-ITD or with *FLT3*-ITD low	Mutated *NPM1* without *FLT3*-ITD (and without adverse-risk cytogenetics)	*FLT3*-ITD allelic ratio is no longer considered due to the impact of midostaurin-based regimens and the absence of a standardized assay to assess it
Biallelic mutated *CEBPA*	bZIP in-frame mutated *CEBPA*	Mono- or biallelic mutational state lost its prognostic weight in the latter classification, with inframe bZIP mutations gaining a predominant role
**Intermediate Risk**		
Mutated *NPM1* with *FLT3*-ITD high	Mutated *NPM1* with *FLT3*-ITD (and without adverse-risk cytogenetics)	
Wild-type *NPM1* without *FLT3*-ITD or with *FLT3*-ITD low (without adverse-risk genetic lesions)	Wild-type *NPM1* with *FLT3*-ITD (without adverse-risk genetic lesions or favorable cytogenetics)	*FLT3*-ITD showed an independent prognostic impact, globally placing patients at intermediate risk
t(9;11)(p21.3;q23.3)/*MLLT3::KMT2A*	t(9;11)(p21.3;q23.3)/*MLLT3::KMT2A*	
Cytogenetic abnormalities not classified as favorable or adverse	Cytogenetic abnormalities not classified as favorable or adverse	
**Adverse Risk**		
t(6;9)(p23;q34.1); *DEK::NUP214*	t(6;9)(p23;q34.1); *DEK::NUP214*	
t(v;11q23.3); *KMT2A* rearranged	t(v;11q23.3); *KMT2A*-rearranged	
t(9;22)(q34.1;q11.2); *BCR::ABL1*	t(9;22)(q34.1;q11.2); *BCR::ABL1*	
	t(8;16)(p11.2;p13.3)/*KAT6A::CREBBP*	New cytogenetic abnormality included in the ELN 2022 classification
inv(3)(q21.3q26.2) or t(3;3)(q21.3;q26.2); *GATA2*, *MECOM*(*EVI1*)	inv(3)(q21.3q26.2) or t(3;3)(q21.3;q26.2)/*GATA2*, *MECOM*(*EVI1*)	
	t(3q26.2;v)/*MECOM*(*EVI1*)-rearranged	New cytogenetic abnormality included in the ELN 2022 classification
−5 or del(5q); −7; −17/abn(17p)	−5 or del(5q); −7; −17/abn(17p)	
Complex karyotype, monosomal karyotype	Complex karyotype, monosomal karyotype	Multiple trisomies or polysomies no longer define CK
Mutated *RUNX1*, *ASXL1*	Mutated *ASXL1*, *BCOR*, *EZH2*, *RUNX1*, *SF3B1*, *SRSF2*, *STAG2*, *U2AF1*, and/or *ZRSR2*	Additional gene mutations are added, irrespective of prior MDS history
Mutated *TP53*	Mutated *TP53*	At least a 10% VAF is required to classify patients as *TP53*-mutated
Wild-type *NPM1* and *FLT3*-ITD high		*FLT3*-ITD define an intermediate risk, irrespective of its allelic ratio or concurrent *NPM1* mutations

Abbreviations: ELN, European Leukemia Net; CK, complex karyotype.

## References

[1] Urbino I., Secreto C., Olivi M., Apolito V., D’Ardia S., Frairia C., Giai V., Aydin S., Freilone R., Dellacasa C. (2021). Evolving Therapeutic Approaches for Older Patients with Acute Myeloid Leukemia in 2021. Cancers.

[2] DiNardo C.D., Wei A.H. (2020). How I Treat Acute Myeloid Leukemia in the Era of New Drugs. Blood.

[3] Itzykson R., Cerrano M., Esteve J., Röllig C., Ossenkoppele G.J. (2021). Prognostic Factors in AML. Acute Myeloid Leukemia.

[4] Elgarten C.W., Aplenc R. (2020). Pediatric acute myeloid leukemia: Updates on biology, risk stratification, and therapy. Curr. Opin. Pediatr..

[5] Sasaki K., Ravandi F., Kadia T.M., DiNardo C.D., Short N.J., Borthakur G., Jabbour E., Kantarjian H.M. (2021). De Novo Acute Myeloid Leukemia: A Population-Based Study of Outcome in the United States Based on the Surveillance, Epidemiology, and End Results (SEER) Database, 1980 to 2017. Cancer.

[6] Lazarevic V., Hörstedt A.-S., Johansson B., Antunovic P., Billström R., Derolf Å., Hulegårdh E., Lehmann S., Möllgård L., Nilsson C. (2014). Incidence and Prognostic Significance of Karyotypic Subgroups in Older Patients with Acute Myeloid Leukemia: The Swedish Population-Based Experience. Blood Cancer J..

[7] Appelbaum F.R., Gundacker H., Head D.R., Slovak M.L., Willman C.L., Godwin J.E., Anderson J.E., Petersdorf S.H. (2006). Age and Acute Myeloid Leukemia. Blood.

[8] Juliusson G., Antunovic P., Derolf A., Lehmann S., Möllgård L., Stockelberg D., Tidefelt U., Wahlin A., Höglund M. (2009). Age and Acute Myeloid Leukemia: Real World Data on Decision to Treat and Outcomes from the Swedish Acute Leukemia Registry. Blood.

[9] Palmieri R., Maurillo L., Del Principe M.I., Paterno G., Walter R.B., Venditti A., Buccisano F. (2022). Time for Dynamic Assessment of Fitness in Acute Myeloid Leukemia. Cancers.

[10] Ferrara F., Barosi G., Venditti A., Angelucci E., Gobbi M., Pane F., Tosi P., Zinzani P., Tura S. (2013). Consensus-Based Definition of Unfitness to Intensive and Non-Intensive Chemotherapy in Acute Myeloid Leukemia: A Project of SIE, SIES and GITMO Group on a New Tool for Therapy Decision Making. Leukemia.

[11] Graf I., Greiner G., Marculescu R., Gleixner K.V., Herndlhofer S., Stefanzl G., Knoebl P., Jäger U., Hauswirth A., Schwarzinger I. (2023). N-Terminal pro-Brain Natriuretic Peptide Is a Prognostic Marker for Response to Intensive Chemotherapy, Early Death, and Overall Survival in Acute Myeloid Leukemia. Am. J. Hematol..

[12] Min G.-J., Cho B.-S., Park S.-S., Park S., Jeon Y.-W., Shin S.-H., Yahng S.-A., Yoon J.-H., Lee S.-E., Eom K.-S. (2022). Geriatric Assessment Predicts Nonfatal Toxicities and Survival for Intensively Treated Older Adults with AML. Blood.

[13] Aydin S., Passera R., Cerrano M., Giai V., D’Ardia S., Iovino G., Dellacasa C.M., Audisio E., Busca A. (2023). Combining the HCT-CI, G8, and AML-Score for Fitness Evaluation of Elderly Patients with Acute Myeloid Leukemia: A Single Center Analysis. Cancers.

[14] Ganzel C., Becker J., Mintz P.D., Lazarus H.M., Rowe J.M. (2012). Hyperleukocytosis, Leukostasis and Leukapheresis: Practice Management. Blood Rev..

[15] Cerrano M., Seegers V., Raffoux E., Rabian F., Sébert M., Itzykson R., Lemiale V., Adès L., Boissel N., Dombret H. (2020). Predictors and Outcomes Associated with Hydroxyurea Sensitivity in Acute Myeloid Leukemia Patients with High Hyperleukocytosis. Leuk Lymphoma.

[16] Frairia C., Nicolino B., Secreto C., Messa E., Arrigo G., Busca A., Cerrano M., D’Ardìa S., Dellacasa C., Evangelista A. (2023). Validation of National Early Warning Score and Quick Sequential (Sepsis-Related) Organ Failure Assessment in Acute Myeloid Leukaemia Patients Treated with Intensive Chemotherapy. Eur. J. Haematol..

[17] Cerrano M., Chevret S., Raffoux E., Rabian F., Sébert M., Valade S., Itzykson R., Lemiale V., Adès L., Boissel N. (2023). Benefits of Dexamethasone on Early Outcomes in Patients with Acute Myeloid Leukemia with Hyperleukocytosis: A Propensity Score Matched Analysis. Ann. Hematol..

[18] Canaani J., Labopin M., Socié G., Nihtinen A., Huynh A., Cornelissen J., Deconinck E., Gedde-Dahl T., Forcade E., Chevallier P. (2017). Long Term Impact of Hyperleukocytosis in Newly Diagnosed Acute Myeloid Leukemia Patients Undergoing Allogeneic Stem Cell Transplantation: An Analysis from the Acute Leukemia Working Party of the EBMT. Am. J. Hematol..

[19] Tien F.-M., Hou H.-A., Tsai C.-H., Tang J.-L., Chen C.-Y., Kuo Y.-Y., Li C.-C., Lin C.-T., Yao M., Huang S.-Y. (2018). Hyperleukocytosis Is Associated with Distinct Genetic Alterations and Is an Independent Poor-Risk Factor in de novo Acute Myeloid Leukemia Patients. Eur. J. Haematol..

[20] Papaemmanuil E., Gerstung M., Bullinger L., Gaidzik V.I., Paschka P., Roberts N.D., Potter N.E., Heuser M., Thol F., Bolli N. (2016). Genomic Classification and Prognosis in Acute Myeloid Leukemia. N. Engl. J. Med..

[21] Ganzel C., Manola J., Douer D., Rowe J.M., Fernandez H.F., Paietta E.M., Litzow M.R., Lee J.-W., Luger S.M., Lazarus H.M. (2016). Extramedullary Disease in Adult Acute Myeloid Leukemia Is Common but Lacks Independent Significance: Analysis of Patients in ECOG-ACRIN Cancer Research Group Trials, 1980–2008. J. Clin. Oncol..

[22] Khoury J.D., Solary E., Abla O., Akkari Y., Alaggio R., Apperley J.F., Bejar R., Berti E., Busque L., Chan J.K.C. (2022). The 5th Edition of the World Health Organization Classification of Haematolymphoid Tumours: Myeloid and Histiocytic/Dendritic Neoplasms. Leukemia.

[23] Granfeldt Østgård L.S., Medeiros B.C., Sengeløv H., Nørgaard M., Andersen M.K., Dufva I.H., Friis L.S., Kjeldsen E., Marcher C.W., Preiss B. (2015). Epidemiology and Clinical Significance of Secondary and Therapy-Related Acute Myeloid Leukemia: A National Population-Based Cohort Study. J. Clin. Oncol..

[24] Schmaelter A.-K., Labopin M., Socié G., Itälä-Remes M., Blaise D., Yakoub-Agha I., Forcade E., Cornelissen J., Ganser A., Beelen D. (2020). Inferior Outcome of Allogeneic Stem Cell Transplantation for Secondary Acute Myeloid Leukemia in First Complete Remission as Compared to de Novo Acute Myeloid Leukemia. Blood Cancer J..

[25] Chanswangphuwana C., Polprasert C., Owattanapanich W., Kungwankiattichai S., Tantiworawit A., Rattanathammethee T., Limvorapitak W., Saengboon S., Niparuck P., Puavilai T. (2022). Characteristics and Outcomes of Secondary Acute Myeloid Leukemia and Acute Myeloid Leukemia with Myelodysplasia-Related Changes: Multicenter Study from the Thai Acute Leukemia Study Group. Clin. Lymphoma Myeloma Leuk..

[26] Higgins A., Shah M.V. (2020). Genetic and Genomic Landscape of Secondary and Therapy-Related Acute Myeloid Leukemia. Genes.

[27] Lindsley R.C., Mar B.G., Mazzola E., Grauman P.V., Shareef S., Allen S.L., Pigneux A., Wetzler M., Stuart R.K., Erba H.P. (2015). Acute Myeloid Leukemia Ontogeny Is Defined by Distinct Somatic Mutations. Blood.

[28] Gurnari C., Fabiani E., Falconi G., Travaglini S., Ottone T., Cristiano A., Voso M.T. (2021). From Clonal Hematopoiesis to Therapy-Related Myeloid Neoplasms: The Silent Way of Cancer Progression. Biology.

[29] Gurnari C., Pagliuca S., Prata P.H., Galimard J.-E., Catto L.F.B., Larcher L., Sebert M., Allain V., Patel B.J., Durmaz A. (2023). Clinical and Molecular Determinants of Clonal Evolution in Aplastic Anemia and Paroxysmal Nocturnal Hemoglobinuria. J. Clin. Oncol..

[30] Schoch C., Kern W., Schnittger S., Hiddemann W., Haferlach T. (2004). Karyotype Is an Independent Prognostic Parameter in Therapy-Related Acute Myeloid Leukemia (t-AML): An Analysis of 93 Patients with t-AML in Comparison to 1091 Patients with de Novo AML. Leukemia.

[31] Ossenkoppele G., Montesinos P. (2019). Challenges in the Diagnosis and Treatment of Secondary Acute Myeloid Leukemia. Crit. Rev. Oncol. Hematol..

[32] Rogers H.J., Wang X., Xie Y., Davis A.R., Thakral B., Wang S.A., Borthakur G., Cantu M.D., Margolskee E.M., Philip J.K.S. (2020). Comparison of Therapy-Related and de Novo Core Binding Factor Acute Myeloid Leukemia: A Bone Marrow Pathology Group Study. Am. J. Hematol..

[33] Nilsson C., Linde F., Hulegårdh E., Garelius H., Lazarevic V., Antunovic P., Cammenga J., Deneberg S., Eriksson A., Jädersten M. (2023). Characterization of Therapy-Related Acute Myeloid Leukemia: Increasing Incidence and Prognostic Implications. Haematologica.

[34] Braun T., Cereja S., Chevret S., Raffoux E., Beaumont M., Detourmignies L., Pigneux A., Thomas X., Bordessoule D., Guerci A. (2015). Evolving Characteristics and Outcome of Secondary Acute Promyelocytic Leukemia (APL): A Prospective Analysis by the French-Belgian-Swiss APL Group. Cancer.

[35] Heuser M. (2016). Therapy-Related Myeloid Neoplasms: Does Knowing the Origin Help to Guide Treatment?. Hematol. Am. Soc. Hematol. Educ. Program.

[36] Rücker F.G., Schlenk R.F., Bullinger L., Kayser S., Teleanu V., Kett H., Habdank M., Kugler C.-M., Holzmann K., Gaidzik V.I. (2012). TP53 Alterations in Acute Myeloid Leukemia with Complex Karyotype Correlate with Specific Copy Number Alterations, Monosomal Karyotype, and Dismal Outcome. Blood.

[37] Hochman M.J., Othus M., Hasserjian R.P., Ambinder A.J., Brunner A.M., Percival M.-E.M., Hourigan C.S., Swords R., DeZern A.E., Estey E.H. (2022). Prognostic Impact of Secondary Versus De Novo Ontogeny in Acute Myeloid Leukemia (AML) Is Predominantly Accounted for By the European Leukemianet (ELN) 2022 Risk Classification. Blood.

[38] Arber D.A., Orazi A., Hasserjian R.P., Borowitz M.J., Calvo K.R., Kvasnicka H.-M., Wang S.A., Bagg A., Barbui T., Branford S. (2022). International Consensus Classification of Myeloid Neoplasms and Acute Leukemias: Integrating Morphologic, Clinical, and Genomic Data. Blood.

[39] Döhner H., Wei A.H., Appelbaum F.R., Craddock C., DiNardo C.D., Dombret H., Ebert B.L., Fenaux P., Godley L.A., Hasserjian R.P. (2022). Diagnosis and Management of AML in Adults: 2022 Recommendations from an International Expert Panel on Behalf of the ELN. Blood.

[40] Boddu P., Kantarjian H.M., Garcia-Manero G., Ravandi F., Verstovsek S., Jabbour E., Borthakur G., Konopleva M., Bhalla K.N., Daver N. (2017). Treated Secondary Acute Myeloid Leukemia: A Distinct High-Risk Subset of AML with Adverse Prognosis. Blood Adv..

[41] Grimwade D., Hills R.K., Moorman A.V., Walker H., Chatters S., Goldstone A.H., Wheatley K., Harrison C.J., Burnett A.K. (2010). On behalf of the National Cancer Research Institute Adult Leukaemia Working Group Refinement of Cytogenetic Classification in Acute Myeloid Leukemia: Determination of Prognostic Significance of Rare Recurring Chromosomal Abnormalities among 5876 Younger Adult Patients Treated in the United Kingdom Medical Research Council Trials. Blood.

[42] Slovak M.L., Kopecky K.J., Cassileth P.A., Harrington D.H., Theil K.S., Mohamed A., Paietta E., Willman C.L., Head D.R., Rowe J.M. (2000). Karyotypic Analysis Predicts Outcome of Preremission and Postremission Therapy in Adult Acute Myeloid Leukemia: A Southwest Oncology Group/Eastern Cooperative Oncology Group Study. Blood.

[43] Byrd J.C., Mrózek K., Dodge R.K., Carroll A.J., Edwards C.G., Arthur D.C., Pettenati M.J., Patil S.R., Rao K.W., Watson M.S. (2002). Pretreatment Cytogenetic Abnormalities Are Predictive of Induction Success, Cumulative Incidence of Relapse, and Overall Survival in Adult Patients with de Novo Acute Myeloid Leukemia: Results from Cancer and Leukemia Group B (CALGB 8461)Presented in Part at the 43rd Annual Meeting of the American Society of Hematology, Orlando, FL, December 10, 2001, and Published in Abstract Form.59. Blood.

[44] Sanz M.A., Fenaux P., Tallman M.S., Estey E.H., Löwenberg B., Naoe T., Lengfelder E., Döhner H., Burnett A.K., Chen S.-J. (2019). Management of Acute Promyelocytic Leukemia: Updated Recommendations from an Expert Panel of the European LeukemiaNet. Blood.

[45] Kuykendall A., Duployez N., Boissel N., Lancet J.E., Welch J.S. (2018). Acute Myeloid Leukemia: The Good, the Bad, and the Ugly. Am. Soc. Clin. Oncol. Educ. Book.

[46] Grimwade D., Mrózek K. (2011). Diagnostic and Prognostic Value of Cytogenetics in Acute Myeloid Leukemia. Hematol./Oncol. Clin. N. Am..

[47] Jourdan E., Boissel N., Chevret S., Delabesse E., Renneville A., Cornillet P., Blanchet O., Cayuela J.-M., Recher C., Raffoux E. (2013). Prospective Evaluation of Gene Mutations and Minimal Residual Disease in Patients with Core Binding Factor Acute Myeloid Leukemia. Blood.

[48] Opatz S., Bamopoulos S.A., Metzeler K.H., Herold T., Ksienzyk B., Bräundl K., Tschuri S., Vosberg S., Konstandin N.P., Wang C. (2020). The Clinical Mutatome of Core Binding Factor Leukemia. Leukemia.

[49] Cher C.Y., Leung G.M.K., Au C.H., Chan T.L., Ma E.S.K., Sim J.P.Y., Gill H., Lie A.K.W., Liang R., Wong K.F. (2016). Next-Generation Sequencing with a Myeloid Gene Panel in Core-Binding Factor AML Showed KIT Activation Loop and TET2 Mutations Predictive of Outcome. Blood Cancer J..

[50] Han S.Y., Mrózek K., Voutsinas J., Wu Q., Morgan E.A., Vestergaard H., Ohgami R., Kluin P.M., Kristensen T.K., Pullarkat S. (2021). Secondary Cytogenetic Abnormalities in Core-Binding Factor AML Harboring Inv(16) vs t(8;21). Blood Adv..

[51] Schlenk R.F., Benner A., Krauter J., Büchner T., Sauerland C., Ehninger G., Schaich M., Mohr B., Niederwieser D., Krahl R. (2004). Individual Patient Data-Based Meta-Analysis of Patients Aged 16 to 60 Years with Core Binding Factor Acute Myeloid Leukemia: A Survey of the German Acute Myeloid Leukemia Intergroup. J. Clin. Oncol..

[52] Herold T., Rothenberg-Thurley M., Grunwald V.V., Janke H., Goerlich D., Sauerland M.C., Konstandin N.P., Dufour A., Schneider S., Neusser M. (2020). Validation and Refinement of the Revised 2017 European LeukemiaNet Genetic Risk Stratification of Acute Myeloid Leukemia. Leukemia.

[53] Boddu P., Gurguis C., Sanford D., Cortes J., Akosile M., Ravandi F., Garcia-Manero G., Patel K.P., Kadia T., Brandt M. (2018). Response Kinetics and Factors Predicting Survival in Core-Binding Factor Leukemia. Leukemia.

[54] Ishikawa Y., Kawashima N., Atsuta Y., Sugiura I., Sawa M., Dobashi N., Yokoyama H., Doki N., Tomita A., Kiguchi T. (2020). Prospective Evaluation of Prognostic Impact of KIT Mutations on Acute Myeloid Leukemia with *RUNX1-RUNX1T1* and *CBFB-MYH11*. Blood Adv..

[55] Paschka P., Du J., Schlenk R.F., Gaidzik V.I., Bullinger L., Corbacioglu A., Späth D., Kayser S., Schlegelberger B., Krauter J. (2013). Secondary Genetic Lesions in Acute Myeloid Leukemia with Inv(16) or t(16;16): A Study of the German-Austrian AML Study Group (AMLSG). Blood.

[56] Christen F., Hoyer K., Yoshida K., Hou H.-A., Waldhueter N., Heuser M., Hills R.K., Chan W., Hablesreiter R., Blau O. (2019). Genomic Landscape and Clonal Evolution of Acute Myeloid Leukemia with t(8;21): An International Study on 331 Patients. Blood.

[57] Tazi Y., Arango-Ossa J.E., Zhou Y., Bernard E., Thomas I., Gilkes A., Freeman S., Pradat Y., Johnson S.J., Hills R. (2022). Unified Classification and Risk-Stratification in Acute Myeloid Leukemia. Nat. Commun..

[58] Boissel N., Leroy H., Brethon B., Philippe N., de Botton S., Auvrignon A., Raffoux E., Leblanc T., Thomas X., Hermine O. (2006). Incidence and Prognostic Impact of *C-Kit*, *FLT3*, and *Ras* Gene Mutations in Core Binding Factor Acute Myeloid Leukemia (CBF-AML). Leukemia.

[59] Paschka P., Marcucci G., Ruppert A.S., Mrózek K., Chen H., Kittles R.A., Vukosavljevic T., Perrotti D., Vardiman J.W., Carroll A.J. (2006). Adverse Prognostic Significance of KIT Mutations in Adult Acute Myeloid Leukemia with Inv(16) and t(8;21): A Cancer and Leukemia Group B Study. J. Clin. Oncol..

[60] Rücker F.G., Agrawal M., Corbacioglu A., Weber D., Kapp-Schwoerer S., Gaidzik V.I., Jahn N., Schroeder T., Wattad M., Lübbert M. (2019). Measurable Residual Disease Monitoring in Acute Myeloid Leukemia with t(8;21)(Q22;Q22.1): Results from the AML Study Group. Blood.

[61] Chen W., Xie H., Wang H., Chen L., Sun Y., Chen Z., Li Q. (2016). Prognostic Significance of KIT Mutations in Core-Binding Factor Acute Myeloid Leukemia: A Systematic Review and Meta-Analysis. PLoS ONE.

[62] Klein K., Kaspers G., Harrison C.J., Beverloo H.B., Reedijk A., Bongers M., Cloos J., Pession A., Reinhardt D., Zimmerman M. (2015). Clinical Impact of Additional Cytogenetic Aberrations, *CKIT* and *RAS* Mutations, and Treatment Elements in Pediatric t(8;21)-AML: Results From an International Retrospective Study by the International Berlin-Frankfurt-Münster Study Group. J. Clin. Oncol..

[63] Itzykson R., Duployez N., Fasan A., Decool G., Marceau-Renaut A., Meggendorfer M., Jourdan E., Petit A., Lapillonne H., Micol J.-B. (2018). Clonal Interference of Signaling Mutations Worsens Prognosis in Core-Binding Factor Acute Myeloid Leukemia. Blood.

[64] Kayser S., Kramer M., Martínez-Cuadrón D., Grenet J., Metzeler K.H., Sustkova Z., Luskin M.R., Brunner A.M., Elliott M.A., Gil C. (2022). Characteristics and Outcome of Patients with Core-Binding Factor Acute Myeloid Leukemia and FLT3-ITD: Results from an International Collaborative Study. Haematologica.

[65] Santos F.P.S., Jones D., Qiao W., Cortes J.E., Ravandi F., Estey E.E., Verma D., Kantarjian H., Borthakur G. (2011). Prognostic Value of *FLT3* Mutations among Different Cytogenetic Subgroups in Acute Myeloid Leukemia. Cancer.

[66] Meyer C., Burmeister T., Gröger D., Tsaur G., Fechina L., Renneville A., Sutton R., Venn N.C., Emerenciano M., Pombo-de-Oliveira M.S. (2018). The MLL Recombinome of Acute Leukemias in 2017. Leukemia.

[67] Tarlock K., Alonzo T.A., Moraleda P.P., Gerbing R.B., Raimondi S.C., Hirsch B.A., Ravindranath Y., Lange B., Woods W.G., Gamis A.S. (2014). Acute Myeloid Leukaemia (AML) with t(6;9)(P23;Q34) Is Associated with Poor Outcome in Childhood AML Regardless of *FLT3*-ITD Status: A Report from the Children’s Oncology Group. Br. J. Haematol..

[68] Díaz-Beyá M., Labopin M., Maertens J., Alijurf M., Passweg J., Dietrich B., Schouten H., Socié G., Schaap N., Schwerdtfeger R. (2020). Allogeneic Stem Cell Transplantation in AML with t(6;9)(P23;Q34);*DEK-NUP214* Shows a Favourable Outcome When Performed in First Complete Remission. Br. J. Haematol..

[69] Lugthart S., Gröschel S., Beverloo H.B., Kayser S., Valk P.J.M., van Zelderen-Bhola S.L., Jan Ossenkoppele G., Vellenga E., van den Berg-de Ruiter E., Schanz U. (2010). Clinical, Molecular, and Prognostic Significance of WHO Type Inv(3)(Q21q26.2)/t(3;3)(Q21;Q26.2) and Various Other 3q Abnormalities in Acute Myeloid Leukemia. J. Clin. Oncol..

[70] Richard-Carpentier G., Rausch C.R., Sasaki K., Hammond D., Morita K., Takahashi K., Tang G., Kanagal-Shamanna R., Bhalla K., Dinardo C.D. (2023). Characteristics and Clinical Outcomes of Patients with Acute Myeloid Leukemia with Inv(3)(Q21q26.2) or t(3;3)(Q21;Q26.2). Haematologica.

[71] Ottema S., Mulet-Lazaro R., Beverloo H.B., Erpelinck C., van Herk S., van der Helm R., Havermans M., Grob T., Valk P.J.M., Bindels E. (2020). Atypical 3q26/MECOM Rearrangements Genocopy Inv(3)/t(3;3) in Acute Myeloid Leukemia. Blood.

[72] Neuendorff N.R., Burmeister T., Dörken B., Westermann J. (2016). BCR-ABL-Positive Acute Myeloid Leukemia: A New Entity? Analysis of Clinical and Molecular Features. Ann. Hematol..

[73] Neuendorff N.R., Hemmati P., Arnold R., Ihlow J., Dörken B., Müller-Tidow C., Westermann J. (2018). *BCR-ABL*+ Acute Myeloid Leukemia: Are We Always Dealing with a High-Risk Disease?. Blood Adv..

[74] Lazarevic V.L., Labopin M., Depei W., Yakoub-Agha I., Huynh A., Ljungman P., Schaap N., Cornelissen J.J., Maillard N., Pioltelli P. (2018). Relatively Favorable Outcome after Allogeneic Stem Cell Transplantation for *BCR-ABL1*-Positive AML: A Survey from the Acute Leukemia Working Party of the European Society for Blood and Marrow Transplantation (EBMT). Am. J. Hematol..

[75] Zhou Q., Zhao D., Eladl E., Capo-Chichi J.-M., Kim D.D.H., Chang H. (2023). Molecular Genetic Characterization of Philadelphia Chromosome-Positive Acute Myeloid Leukemia. Leuk. Res..

[76] Kayser S., Hills R.K., Langova R., Kramer M., Guijarro F., Sustkova Z., Estey E.H., Shaw C.M., Ráčil Z., Mayer J. (2021). Characteristics and Outcome of Patients with Acute Myeloid Leukaemia and t(8;16)(P11;P13): Results from an International Collaborative Study*. Br. J. Haematol..

[77] Greenberg P.L., Tuechler H., Schanz J., Sanz G., Garcia-Manero G., Solé F., Bennett J.M., Bowen D., Fenaux P., Dreyfus F. (2012). Revised International Prognostic Scoring System for Myelodysplastic Syndromes. Blood.

[78] Creutzig U., Zimmermann M., Reinhardt D., Rasche M., von Neuhoff C., Alpermann T., Dworzak M., Perglerová K., Zemanova Z., Tchinda J. (2016). Changes in Cytogenetics and Molecular Genetics in Acute Myeloid Leukemia from Childhood to Adult Age Groups. Cancer.

[79] Stölzel F., Mohr B., Kramer M., Oelschlägel U., Bochtler T., Berdel W.E., Kaufmann M., Baldus C.D., Schäfer-Eckart K., Stuhlmann R. (2016). Karyotype Complexity and Prognosis in Acute Myeloid Leukemia. Blood Cancer J..

[80] Haferlach C., Alpermann T., Schnittger S., Kern W., Chromik J., Schmid C., Pielken H.J., Kreuzer K.-A., Höffkes H.-G., Haferlach T. (2012). Prognostic Value of Monosomal Karyotype in Comparison to Complex Aberrant Karyotype in Acute Myeloid Leukemia: A Study on 824 Cases with Aberrant Karyotype. Blood.

[81] Schoch C., Kern W., Kohlmann A., Hiddemann W., Schnittger S., Haferlach T. (2005). Acute Myeloid Leukemia with a Complex Aberrant Karyotype Is a Distinct Biological Entity Characterized by Genomic Imbalances and a Specific Gene Expression Profile. Genes Chromosomes Cancer.

[82] Mrózek K., Eisfeld A.-K., Kohlschmidt J., Carroll A.J., Walker C.J., Nicolet D., Blachly J.S., Bill M., Papaioannou D., Wang E.S. (2019). Complex Karyotype in de Novo Acute Myeloid Leukemia: Typical and Atypical Subtypes Differ Molecularly and Clinically. Leukemia.

[83] Breems D.A., Van Putten W.L.J., De Greef G.E., Van Zelderen-Bhola S.L., Gerssen-Schoorl K.B.J., Mellink C.H.M., Nieuwint A., Jotterand M., Hagemeijer A., Beverloo H.B. (2008). Monosomal Karyotype in Acute Myeloid Leukemia: A Better Indicator of Poor Prognosis than a Complex Karyotype. J. Clin. Oncol..

[84] Medeiros B.C., Othus M., Fang M., Roulston D., Appelbaum F.R. (2010). Prognostic Impact of Monosomal Karyotype in Young Adult and Elderly Acute Myeloid Leukemia: The Southwest Oncology Group (SWOG) Experience. Blood.

[85] Lazarevic V., Rosso A., Juliusson G., Antunovic P., Rangert-Derolf Å., Lehmann S., Möllgård L., Uggla B., Wennström L., Wahlin A. (2015). Prognostic Significance of High Hyperdiploid and Triploid/Tetraploid Adult Acute Myeloid Leukemia. Am. J. Hematol..

[86] Kayser S., Martínez-Cuadrón D., Hanoun M., Stölzel F., Gil C., Reinhardt H.C., Aguiar E., Schäfer-Eckart K., Burgues J.M.B., Steffen B. (2023). Characteristics and Outcome of Patients with Acute Myeloid Leukemia and Trisomy 4. Haematologica.

[87] Chilton L., Hills R.K., Harrison C.J., Burnett A.K., Grimwade D., Moorman A.V. (2014). Hyperdiploidy with 49-65 Chromosomes Represents a Heterogeneous Cytogenetic Subgroup of Acute Myeloid Leukemia with Differential Outcome. Leukemia.

[88] Grimwade D., Ivey A., Huntly B.J.P. (2016). Molecular Landscape of Acute Myeloid Leukemia in Younger Adults and Its Clinical Relevance. Blood.

[89] Whitman S.P., Maharry K., Radmacher M.D., Becker H., Mrózek K., Margeson D., Holland K.B., Wu Y.-Z., Schwind S., Metzeler K.H. (2010). FLT3 Internal Tandem Duplication Associates with Adverse Outcome and Gene- and MicroRNA-Expression Signatures in Patients 60 Years of Age or Older with Primary Cytogenetically Normal Acute Myeloid Leukemia: A Cancer and Leukemia Group B Study. Blood.

[90] Eisfeld A.-K., Kohlschmidt J., Mrózek K., Blachly J.S., Walker C.J., Nicolet D., Orwick S., Maharry S.E., Carroll A.J., Stone R.M. (2018). Mutation Patterns Identify Adult Patients with de Novo Acute Myeloid Leukemia Aged 60 Years or Older Who Respond Favorably to Standard Chemotherapy: An Analysis of Alliance Studies. Leukemia.

[91] Liu S.-B., Dong H.-J., Bao X.-B., Qiu Q.-C., Li H.-Z., Shen H.-J., Ding Z.-X., Wang C., Chu X.-L., Yu J.-Q. (2019). Impact of *FLT3*-ITD Length on Prognosis of Acute Myeloid Leukemia. Haematologica.

[92] Bacher U., Haferlach C., Kern W., Haferlach T., Schnittger S. (2008). Prognostic Relevance of *FLT3*-TKD Mutations in AML: The Combination Matters—An Analysis of 3082 Patients. Blood.

[93] Mead A.J., Linch D.C., Hills R.K., Wheatley K., Burnett A.K., Gale R.E. (2007). *FLT3* Tyrosine Kinase Domain Mutations Are Biologically Distinct from and Have a Significantly More Favorable Prognosis than *FLT3* Internal Tandem Duplications in Patients with Acute Myeloid Leukemia. Blood.

[94] Boddu P., Kantarjian H., Borthakur G., Kadia T., Daver N., Pierce S., Andreeff M., Ravandi F., Cortes J., Kornblau S.M. (2017). Co-Occurrence of *FLT3-TKD* and *NPM1* Mutations Defines a Highly Favorable Prognostic AML Group. Blood Adv..

[95] Falini B., Mecucci C., Tiacci E., Alcalay M., Rosati R., Pasqualucci L., La Starza R., Diverio D., Colombo E., Santucci A. (2005). Cytoplasmic Nucleophosmin in Acute Myelogenous Leukemia with a Normal Karyotype. N. Engl. J. Med..

[96] Thiede C., Koch S., Creutzig E., Steudel C., Illmer T., Schaich M., Ehninger G. (2006). Prevalence and Prognostic Impact of *NPM1* Mutations in 1485 Adult Patients with Acute Myeloid Leukemia (AML). Blood.

[97] Schneider F., Hoster E., Unterhalt M., Schneider S., Dufour A., Benthaus T., Mellert G., Zellmeier E., Kakadia P.M., Bohlander S.K. (2012). The *FLT3ITD* MRNA Level Has a High Prognostic Impact in *NPM1* Mutated, but Not in *NPM1* Unmutated, AML with a Normal Karyotype. Blood.

[98] Patel S.S., Kuo F.C., Gibson C.J., Steensma D.P., Soiffer R.J., Alyea E.P., Chen Y.-B.A., Fathi A.T., Graubert T.A., Brunner A.M. (2018). High *NPM1* Mutant Allele Burden at Diagnosis Predicts Unfavorable Outcomes in de Novo AML. Blood.

[99] Wang Y., Quesada A.E., Zuo Z., Medeiros L.J., Yin C.C., Li S., Xu J., Borthakur G., Li Y., Yang C. (2023). The Impact of Mutation of Myelodysplasia-Related Genes in De Novo Acute Myeloid Leukemia Carrying *NPM1* Mutation. Cancers.

[100] Haferlach C., Mecucci C., Schnittger S., Kohlmann A., Mancini M., Cuneo A., Testoni N., Rege-Cambrin G., Santucci A., Vignetti M. (2009). AML with Mutated *NPM1* Carrying a Normal or Aberrant Karyotype Show Overlapping Biologic, Pathologic, Immunophenotypic, and Prognostic Features. Blood.

[101] Angenendt L., Röllig C., Montesinos P., Martínez-Cuadrón D., Barragan E., García R., Botella C., Martínez P., Ravandi F., Kadia T. (2019). Chromosomal Abnormalities and Prognosis in *NPM1*-Mutated Acute Myeloid Leukemia: A Pooled Analysis of Individual Patient Data From Nine International Cohorts. J. Clin. Oncol..

[102] Othman J., Meggendorfer M., Tiacci E., Thiede C., Schlenk R., Dillon R., Stasik S., Venanzi A., Bertoli S., Delabesse E. (2023). Overlapping Features of Therapy-Related and de Novo *NPM1*-Mutated AML. Blood.

[103] Arber D.A., Orazi A., Hasserjian R., Thiele J., Borowitz M.J., Le Beau M.M., Bloomfield C.D., Cazzola M., Vardiman J.W. (2016). The 2016 Revision to the World Health Organization Classification of Myeloid Neoplasms and Acute Leukemia. Blood.

[104] Schlenk R.F., Döhner K., Krauter J., Fröhling S., Corbacioglu A., Bullinger L., Habdank M., Späth D., Morgan M., Benner A. (2008). Mutations and Treatment Outcome in Cytogenetically Normal Acute Myeloid Leukemia. N. Engl. J. Med..

[105] Fröhling S., Schlenk R.F., Stolze I., Bihlmayr J., Benner A., Kreitmeier S., Tobis K., Döhner H., Döhner K. (2004). CEBPA Mutations in Younger Adults with Acute Myeloid Leukemia and Normal Cytogenetics: Prognostic Relevance and Analysis of Cooperating Mutations. J. Clin. Oncol..

[106] Renneville A., Boissel N., Gachard N., Naguib D., Bastard C., de Botton S., Nibourel O., Pautas C., Reman O., Thomas X. (2009). The Favorable Impact of CEBPA Mutations in Patients with Acute Myeloid Leukemia Is Only Observed in the Absence of Associated Cytogenetic Abnormalities and FLT3 Internal Duplication. Blood.

[107] Taskesen E., Bullinger L., Corbacioglu A., Sanders M.A., Erpelinck C.A.J., Wouters B.J., van der Poel-van de Luytgaarde S.C., Damm F., Krauter J., Ganser A. (2011). Prognostic Impact, Concurrent Genetic Mutations, and Gene Expression Features of AML with *CEBPA* Mutations in a Cohort of 1182 Cytogenetically Normal AML Patients: Further Evidence for *CEBPA* Double Mutant AML as a Distinctive Disease Entity. Blood.

[108] Taube F., Georgi J.A., Kramer M., Stasik S., Middeke J.M., Röllig C., Krug U., Krämer A., Scholl S., Hochhaus A. (2022). *CEBPA* Mutations in 4708 Patients with Acute Myeloid Leukemia: Differential Impact of BZIP and TAD Mutations on Outcome. Blood.

[109] Wakita S., Sakaguchi M., Oh I., Kako S., Toya T., Najima Y., Doki N., Kanda J., Kuroda J., Mori S. (2022). Prognostic Impact of *CEBPA* BZIP Domain Mutation in Acute Myeloid Leukemia. Blood Adv..

[110] Konstandin N.P., Pastore F., Herold T., Dufour A., Rothenberg-Thurley M., Hinrichsen T., Ksienzyk B., Tschuri S., Schneider S., Hoster E. (2018). Genetic Heterogeneity of Cytogenetically Normal AML with Mutations of *CEBPA*. Blood Adv..

[111] Tien F.-M., Hou H.-A., Tang J.-L., Kuo Y.-Y., Chen C.-Y., Tsai C.-H., Yao M., Lin C.-T., Li C.-C., Huang S.-Y. (2018). Concomitant *WT1* Mutations Predict Poor Prognosis in Acute Myeloid Leukemia Patients with Double Mutant *CEBPA*. Haematologica.

[112] Weinberg O.K., Siddon A., Madanat Y.F., Gagan J., Arber D.A., Dal Cin P., Narayanan D., Ouseph M.M., Kurzer J.H., Hasserjian R.P. (2022). *TP53* Mutation Defines a Unique Subgroup within Complex Karyotype de Novo and Therapy-Related MDS/AML. Blood Adv..

[113] Grob T., Al Hinai A.S.A., Sanders M.A., Kavelaars F.G., Rijken M., Gradowska P.L., Biemond B.J., Breems D.A., Maertens J., van Marwijk Kooy M. (2022). Molecular Characterization of Mutant *TP53* Acute Myeloid Leukemia and High-Risk Myelodysplastic Syndrome. Blood.

[114] Dutta S., Moritz J., Pregartner G., Thallinger G.G., Brandstätter I., Lind K., Rezania S., Lyssy F., Reinisch A., Zebisch A. (2022). Comparison of Acute Myeloid Leukemia and Myelodysplastic Syndromes with *TP53* Aberrations. Ann. Hematol..

[115] Bernard E., Nannya Y., Hasserjian R.P., Devlin S.M., Tuechler H., Medina-Martinez J.S., Yoshizato T., Shiozawa Y., Saiki R., Malcovati L. (2020). Implications of *TP53* Allelic State for Genome Stability, Clinical Presentation and Outcomes in Myelodysplastic Syndromes. Nat. Med..

[116] Fenwarth L., Vasseur L., Duployez N., Gardin C., Terré C., Lambert J., de Botton S., Celli-Lebras K., Turlure P., Cluzeau T. (2022). Prognostic Impact of Monoallelic Versus Biallelic *TP53* Alterations in Intensively-Treated Adults AML Patients: A Retrospective Study from the ALFA Group. Blood.

[117] Bahaj W., Kewan T., Gurnari C., Durmaz A., Ponvilawan B., Pandit I., Kubota Y., Ogbue O., Aly M., Madanat Y.F. (2022). A Clinically Practicable Approach to Predict *TP53* Allelic Configurations in Myeloid Neoplasia. Blood.

[118] Short N.J., Montalban-Bravo G., Hwang H., Ning J., Franquiz M.J., Kanagal-Shamanna R., Patel K.P., DiNardo C.D., Ravandi F., Garcia-Manero G. (2020). Prognostic and Therapeutic Impacts of Mutant *TP53* Variant Allelic Frequency in Newly Diagnosed Acute Myeloid Leukemia. Blood Adv..

[119] Kihara R., Nagata Y., Kiyoi H., Kato T., Yamamoto E., Suzuki K., Chen F., Asou N., Ohtake S., Miyawaki S. (2014). Comprehensive Analysis of Genetic Alterations and Their Prognostic Impacts in Adult Acute Myeloid Leukemia Patients. Leukemia.

[120] Gaidzik V.I., Teleanu V., Papaemmanuil E., Weber D., Paschka P., Hahn J., Wallrabenstein T., Kolbinger B., Köhne C.H., Horst H.A. (2016). *RUNX1* Mutations in Acute Myeloid Leukemia Are Associated with Distinct Clinico-Pathologic and Genetic Features. Leukemia.

[121] Greif P.A., Konstandin N.P., Metzeler K.H., Herold T., Pasalic Z., Ksienzyk B., Dufour A., Schneider F., Schneider S., Kakadia P.M. (2012). *RUNX1* Mutations in Cytogenetically Normal Acute Myeloid Leukemia Are Associated with a Poor Prognosis and Up-Regulation of Lymphoid Genes. Haematologica.

[122] Metzeler K.H., Herold T., Rothenberg-Thurley M., Amler S., Sauerland M.C., Görlich D., Schneider S., Konstandin N.P., Dufour A., Bräundl K. (2016). Spectrum and Prognostic Relevance of Driver Gene Mutations in Acute Myeloid Leukemia. Blood.

[123] Schnittger S., Eder C., Jeromin S., Alpermann T., Fasan A., Grossmann V., Kohlmann A., Illig T., Klopp N., Wichmann H.-E. (2013). *ASXL1* Exon 12 Mutations Are Frequent in AML with Intermediate Risk Karyotype and Are Independently Associated with an Adverse Outcome. Leukemia.

[124] Pratcorona M., Abbas S., Sanders M.A., Koenders J.E., Kavelaars F.G., Erpelinck-Verschueren C.A.J., Zeilemakers A., Löwenberg B., Valk P.J.M. (2012). Acquired Mutations in *ASXL1* in Acute Myeloid Leukemia: Prevalence and Prognostic Value. Haematologica.

[125] Döhner H., Estey E., Grimwade D., Amadori S., Appelbaum F.R., Büchner T., Dombret H., Ebert B.L., Fenaux P., Larson R.A. (2017). Diagnosis and Management of AML in Adults: 2017 ELN Recommendations from an International Expert Panel. Blood.

[126] Chiche E., Rahmé R., Bertoli S., Dumas P.-Y., Micol J.-B., Hicheri Y., Pasquier F., Peterlin P., Chevallier P., Thomas X. (2021). Real-Life Experience with CPX-351 and Impact on the Outcome of High-Risk AML Patients: A Multicentric French Cohort. Blood Adv..

[127] Othman J., Wilhelm-Benartzi C.S., Dillon R., Knapper S., Freeman S.D., Batten L.M., Canham J., Hinson E.L., Wych J., Betteridge S. (2023). A Randomised Comparison of CPX-351 and FLAG-Ida in Adverse Karyotype AML and High-Risk MDS: The UK NCRI AML19 Trial. Blood Adv..

[128] Kayser S., Levis M.J. (2023). The Clinical Impact of the Molecular Landscape of Acute Myeloid Leukemia. Haematologica.

[129] Bullinger L., Döhner K., Döhner H. (2017). Genomics of Acute Myeloid Leukemia Diagnosis and Pathways. J. Clin. Oncol..

[130] Ley T.J., Ding L., Walter M.J., McLellan M.D., Lamprecht T., Larson D.E., Kandoth C., Payton J.E., Baty J., Welch J. (2010). DNMT3A Mutations in Acute Myeloid Leukemia. N. Engl. J. Med..

[131] Thol F., Damm F., Lüdeking A., Winschel C., Wagner K., Morgan M., Yun H., Göhring G., Schlegelberger B., Hoelzer D. (2011). Incidence and Prognostic Influence of *DNMT3A* Mutations in Acute Myeloid Leukemia. J. Clin. Oncol..

[132] Ahn J.-S., Kim H.-J., Kim Y.-K., Lee S.-S., Jung S.-H., Yang D.-H., Lee J.-J., Kim N.Y., Choi S.H., Jung C.W. (2016). *DNMT3A R882* Mutation with *FLT3-ITD* Positivity Is an Extremely Poor Prognostic Factor in Patients with Normal-Karyotype Acute Myeloid Leukemia after Allogeneic Hematopoietic Cell Transplantation. Biol. Blood Marrow Transplant..

[133] Vetro C., Haferlach T., Meggendorfer M., Stengel A., Jeromin S., Kern W., Haferlach C. (2020). Cytogenetic and Molecular Genetic Characterization of *KMT2A-PTD* Positive Acute Myeloid Leukemia in Comparison to *KMT2A*-Rearranged Acute Myeloid Leukemia. Cancer Genet..

[134] Hinai A.S.A.A., Pratcorona M., Grob T., Kavelaars F.G., Bussaglia E., Sanders M.A., Nomdedeu J., Valk P.J.M. (2019). The Landscape of *KMT2A*-PTD AML: Concurrent Mutations, Gene Expression Signatures, and Clinical Outcome. Hemasphere.

[135] Paschka P., Marcucci G., Ruppert A.S., Whitman S.P., Mrózek K., Maharry K., Langer C., Baldus C.D., Zhao W., Powell B.L. (2008). Wilms’ Tumor 1 Gene Mutations Independently Predict Poor Outcome in Adults with Cytogenetically Normal Acute Myeloid Leukemia: A Cancer and Leukemia Group B Study. J. Clin. Oncol..

[136] Gaidzik V.I., Schlenk R.F., Moschny S., Becker A., Bullinger L., Corbacioglu A., Krauter J., Schlegelberger B., Ganser A., Döhner H. (2009). Prognostic Impact of WT1 Mutations in Cytogenetically Normal Acute Myeloid Leukemia: A Study of the German-Austrian AML Study Group. Blood.

[137] Sargas C., Ayala R., Larráyoz M.J., Chillón M.C., Carrillo-Cruz E., Bilbao-Sieyro C., Prados de la Torre E., Martínez-Cuadrón D., Rodríguez-Veiga R., Boluda B. (2023). Molecular Landscape and Validation of New Genomic Classification in 2668 Adult AML Patients: Real Life Data from the PETHEMA Registry. Cancers.

[138] Alfayez M., Issa G.C., Patel K.P., Wang F., Wang X., Short N.J., Cortes J.E., Kadia T., Ravandi F., Pierce S. (2021). The Clinical Impact of *PTPN11* Mutations in Adults with Acute Myeloid Leukemia. Leukemia.

[139] Stasik S., Eckardt J.-N., Kramer M., Röllig C., Krämer A., Scholl S., Hochhaus A., Crysandt M., Brümmendorf T.H., Naumann R. (2021). Impact of *PTPN11* Mutations on Clinical Outcome Analyzed in 1529 Patients with Acute Myeloid Leukemia. Blood Adv..

[140] Fobare S., Kohlschmidt J., Ozer H.G., Mrózek K., Nicolet D., Mims A.S., Garzon R., Blachly J.S., Orwick S., Carroll A.J. (2022). Molecular, Clinical, and Prognostic Implications of *PTPN11* Mutations in Acute Myeloid Leukemia. Blood Adv..

[141] Haferlach T., Kohlmann A., Klein H.-U., Ruckert C., Dugas M., Williams P.M., Kern W., Schnittger S., Bacher U., Löffler H. (2009). AML with Translocation t(8;16)(P11;P13) Demonstrates Unique Cytomorphological, Cytogenetic, Molecular and Prognostic Features. Leukemia.

[142] Lamble A.J., Hagiwara K., Gerbing R.B., Smith J.L., Kolekar P., Ries R.E., Kolb E.A., Alonzo T.A., Ma X., Meshinchi S. (2023). *CREBBP* Alterations Are Associated with a Poor Prognosis in *de Novo* AML. Blood.

[143] Sébert M., Passet M., Raimbault A., Rahmé R., Raffoux E., Sicre de Fontbrune F., Cerrano M., Quentin S., Vasquez N., Da Costa M. (2019). Germline DDX41 mutations define a significant entity within adult MDS/AML patients. Blood.

[144] Duployez N., Largeaud L., Duchmann M., Kim R., Rieunier J., Lambert J., Bidet A., Larcher L., Lemoine J., Delhommeau F. (2022). Prognostic Impact of *DDX41* Germline Mutations in Intensively Treated Acute Myeloid Leukemia Patients: An ALFA-FILO Study. Blood.

[145] Bassan R., Brüggemann M., Radcliffe H.-S., Hartfield E., Kreuzbauer G., Wetten S. (2019). A Systematic Literature Review and Meta-Analysis of Minimal Residual Disease as a Prognostic Indicator in Adult B-Cell Acute Lymphoblastic Leukemia. Haematologica.

[146] Blachly J.S., Walter R.B., Hourigan C.S. (2022). The Present and Future of Measurable Residual Disease Testing in Acute Myeloid Leukemia. Haematologica.

[147] Schuurhuis G.J., Heuser M., Freeman S., Béné M.-C., Buccisano F., Cloos J., Grimwade D., Haferlach T., Hills R.K., Hourigan C.S. (2018). Minimal/Measurable Residual Disease in AML: A Consensus Document from the European LeukemiaNet MRD Working Party. Blood.

[148] Heuser M., Freeman S.D., Ossenkoppele G.J., Buccisano F., Hourigan C.S., Ngai L.L., Tettero J.M., Bachas C., Baer C., Béné M.-C. (2021). 2021 Update on MRD in Acute Myeloid Leukemia: A Consensus Document from the European LeukemiaNet MRD Working Party. Blood.

[149] Bernardi M., Ferrara F., Carrabba M.G., Mastaglio S., Lorentino F., Vago L., Ciceri F. (2022). MRD in Venetoclax-Based Treatment for AML: Does It Really Matter?. Front. Oncol..

[150] Pratz K.W., Jonas B.A., Pullarkat V., Recher C., Schuh A.C., Thirman M.J., Garcia J.S., DiNardo C.D., Vorobyev V., Fracchiolla N.S. (2022). Measurable Residual Disease Response and Prognosis in Treatment-Naïve Acute Myeloid Leukemia with Venetoclax and Azacitidine. J. Clin. Oncol..

[151] Maiti A., DiNardo C.D., Wang S.A., Jorgensen J., Kadia T.M., Daver N.G., Short N.J., Yilmaz M., Pemmaraju N., Borthakur G. (2021). Prognostic Value of Measurable Residual Disease after Venetoclax and Decitabine in Acute Myeloid Leukemia. Blood Adv..

[152] Walter R.B., Buckley S.A., Pagel J.M., Wood B.L., Storer B.E., Sandmaier B.M., Fang M., Gyurkocza B., Delaney C., Radich J.P. (2013). Significance of Minimal Residual Disease before Myeloablative Allogeneic Hematopoietic Cell Transplantation for AML in First and Second Complete Remission. Blood.

[153] Anthias C., Dignan F.L., Morilla R., Morilla A., Ethell M.E., Potter M.N., Shaw B.E. (2014). Pre-Transplant MRD Predicts Outcome Following Reduced-Intensity and Myeloablative Allogeneic Hemopoietic SCT in AML. Bone Marrow Transpl..

[154] Buckley S.A., Wood B.L., Othus M., Hourigan C.S., Ustun C., Linden M.A., DeFor T.E., Malagola M., Anthias C., Valkova V. (2017). Minimal Residual Disease Prior to Allogeneic Hematopoietic Cell Transplantation in Acute Myeloid Leukemia: A Meta-Analysis. Haematologica.

[155] Pollyea D.A., Altman J.K., Assi R., Bixby D., Fathi A.T., Foran J.M., Gojo I., Hall A.C., Jonas B.A., Kishtagari A. (2023). Acute Myeloid Leukemia, Version 3.2023, NCCN Clinical Practice Guidelines in Oncology. J. Natl. Compr. Cancer Netw..

[156] Döhner K., Thiede C., Jahn N., Panina E., Gambietz A., Larson R.A., Prior T.W., Marcucci G., Jones D., Krauter J. (2020). Impact of *NPM1/FLT3*-ITD Genotypes Defined by the 2017 European LeukemiaNet in Patients with Acute Myeloid Leukemia. Blood.

[157] Lachowiez C.A., Long N., Saultz J., Gandhi A., Newell L.F., Hayes-Lattin B., Maziarz R.T., Leonard J., Bottomly D., McWeeney S. (2023). Comparison and Validation of the 2022 European LeukemiaNet Guidelines in Acute Myeloid Leukemia. Blood Adv..

[158] Lo M.-Y., Tsai X.C.-H., Lin C.-C., Tien F.-M., Kuo Y.-Y., Lee W.-H., Peng Y.-L., Liu M.-C., Tseng M.-H., Hsu C.-A. (2023). Validation of the Prognostic Significance of the 2022 European LeukemiaNet Risk Stratification System in Intensive Chemotherapy Treated Aged 18 to 65 Years Patients with de Novo Acute Myeloid Leukemia. Am. J. Hematol..

[159] Rausch C., Rothenberg-Thurley M., Dufour A., Schneider S., Gittinger H., Sauerland C., Görlich D., Krug U., Berdel W.E., Woermann B.J. (2023). Validation and Refinement of the 2022 European LeukemiaNet Genetic Risk Stratification of Acute Myeloid Leukemia. Leukemia.

[160] Mrózek K., Kohlschmidt J., Blachly J.S., Nicolet D., Carroll A.J., Archer K.J., Mims A.S., Larkin K.T., Orwick S., Oakes C.C. (2023). Outcome Prediction by the 2022 European LeukemiaNet Genetic-Risk Classification for Adults with Acute Myeloid Leukemia: An Alliance Study. Leukemia.

[161] Döhner H., Pratz K.W., DiNardo C.D., Jonas B.A., Pullarkat V.A., Thirman M.J., Recher C., Schuh A.C., Babu S., Dail M. (2022). ELN Risk Stratification Is Not Predictive of Outcomes for Treatment-Naïve Patients with Acute Myeloid Leukemia Treated with Venetoclax and Azacitidine. Blood.

[162] Liu Y., Cheng Z., Pang Y., Cui L., Qian T., Quan L., Zhao H., Shi J., Ke X., Fu L. (2019). Role of MicroRNAs, CircRNAs and Long Noncoding RNAs in Acute Myeloid Leukemia. J. Hematol. Oncol..

[163] Li J., Sun C.-K. (2018). Long Noncoding RNA SNHG5 Is Up-Regulated and Serves as a Potential Prognostic Biomarker in Acute Myeloid Leukemia. Eur. Rev. Med. Pharmacol. Sci..

[164] Garzon R., Volinia S., Papaioannou D., Nicolet D., Kohlschmidt J., Yan P.S., Mrózek K., Bucci D., Carroll A.J., Baer M.R. (2014). Expression and Prognostic Impact of LncRNAs in Acute Myeloid Leukemia. Proc. Natl. Acad. Sci. USA.

[165] Beck D., Thoms J.A.I., Palu C., Herold T., Shah A., Olivier J., Boelen L., Huang Y., Chacon D., Brown A. (2018). A Four-Gene LincRNA Expression Signature Predicts Risk in Multiple Cohorts of Acute Myeloid Leukemia Patients. Leukemia.

[166] Farrar J.E., Smith J.L., Othus M., Huang B.J., Wang Y.-C., Ries R., Hylkema T., Pogosova-Agadjanyan E.L., Challa S., Leonti A. (2023). Long Noncoding RNA Expression Independently Predicts Outcome in Pediatric Acute Myeloid Leukemia. J. Clin. Oncol..

[167] Marcucci G., Radmacher M.D., Maharry K., Mrózek K., Ruppert A.S., Paschka P., Vukosavljevic T., Whitman S.P., Baldus C.D., Langer C. (2008). MicroRNA Expression in Cytogenetically Normal Acute Myeloid Leukemia. N. Engl. J. Med..

[168] Marcucci G., Maharry K.S., Metzeler K.H., Volinia S., Wu Y.-Z., Mrózek K., Nicolet D., Kohlschmidt J., Whitman S.P., Mendler J.H. (2013). Clinical Role of MicroRNAs in Cytogenetically Normal Acute Myeloid Leukemia: MiR-155 Upregulation Independently Identifies High-Risk Patients. J. Clin. Oncol..

[169] Díaz-Beyá M., Brunet S., Nomdedéu J., Tejero R., Díaz T., Pratcorona M., Tormo M., Ribera J.M., Escoda L., Duarte R. (2014). MicroRNA Expression at Diagnosis Adds Relevant Prognostic Information to Molecular Categorization in Patients with Intermediate-Risk Cytogenetic Acute Myeloid Leukemia. Leukemia.

[170] L′Abbate A., Tolomeo D., Cifola I., Severgnini M., Turchiano A., Augello B., Squeo G., D′Addabbo P., Traversa D., Daniele G. (2018). *MYC*-Containing Amplicons in Acute Myeloid Leukemia: Genomic Structures, Evolution, and Transcriptional Consequences. Leukemia.

[171] Figueroa M.E., Lugthart S., Li Y., Erpelinck-Verschueren C., Deng X., Christos P.J., Schifano E., Booth J., van Putten W., Skrabanek L. (2010). DNA Methylation Signatures Identify Biologically Distinct Subtypes in Acute Myeloid Leukemia. Cancer Cell.

[172] Bullinger L., Ehrich M., Döhner K., Schlenk R.F., Döhner H., Nelson M.R., van den Boom D. (2010). Quantitative DNA Methylation Predicts Survival in Adult Acute Myeloid Leukemia. Blood.

[173] Marcucci G., Yan P., Maharry K., Frankhouser D., Nicolet D., Metzeler K.H., Kohlschmidt J., Mrózek K., Wu Y.-Z., Bucci D. (2014). Epigenetics Meets Genetics in Acute Myeloid Leukemia: Clinical Impact of a Novel Seven-Gene Score. J. Clin. Oncol..

[174] Deneberg S., Guardiola P., Lennartsson A., Qu Y., Gaidzik V., Blanchet O., Karimi M., Bengtzén S., Nahi H., Uggla B. (2011). Prognostic DNA Methylation Patterns in Cytogenetically Normal Acute Myeloid Leukemia Are Predefined by Stem Cell Chromatin Marks. Blood.

[175] Jost E., Lin Q., Weidner C.I., Wilop S., Hoffmann M., Walenda T., Schemionek M., Herrmann O., Zenke M., Brümmendorf T.H. (2014). Epimutations Mimic Genomic Mutations of *DNMT3A* in Acute Myeloid Leukemia. Leukemia.

[176] Luskin M.R., Gimotty P.A., Smith C., Loren A.W., Figueroa M.E., Harrison J., Sun Z., Tallman M.S., Paietta E.M., Litzow M.R. (2016). A Clinical Measure of DNA Methylation Predicts Outcome in de Novo Acute Myeloid Leukemia. JCI Insight.

[177] DiNardo C.D., Luskin M.R., Carroll M., Smith C., Harrison J., Pierce S., Kornblau S., Konopleva M., Kadia T., Kantarjian H. (2017). Validation of a Clinical Assay of Multi-Locus DNA Methylation for Prognosis of Newly Diagnosed AML. Am. J. Hematol..

[178] Metzeler K.H., Hummel M., Bloomfield C.D., Spiekermann K., Braess J., Sauerland M.-C., Heinecke A., Radmacher M., Marcucci G., Whitman S.P. (2008). An 86-Probe-Set Gene-Expression Signature Predicts Survival in Cytogenetically Normal Acute Myeloid Leukemia. Blood.

[179] Levine J.H., Simonds E.F., Bendall S.C., Davis K.L., Amir E.D., Tadmor M.D., Litvin O., Fienberg H.G., Jager A., Zunder E.R. (2015). Data-Driven Phenotypic Dissection of AML Reveals Progenitor-like Cells That Correlate with Prognosis. Cell.

[180] Eppert K., Takenaka K., Lechman E.R., Waldron L., Nilsson B., van Galen P., Metzeler K.H., Poeppl A., Ling V., Beyene J. (2011). Stem Cell Gene Expression Programs Influence Clinical Outcome in Human Leukemia. Nat. Med..

[181] Ng S.W.K., Mitchell A., Kennedy J.A., Chen W.C., McLeod J., Ibrahimova N., Arruda A., Popescu A., Gupta V., Schimmer A.D. (2016). A 17-Gene Stemness Score for Rapid Determination of Risk in Acute Leukaemia. Nature.

[182] Duployez N., Marceau-Renaut A., Villenet C., Petit A., Rousseau A., Ng S.W.K., Paquet A., Gonzales F., Barthélémy A., Leprêtre F. (2019). The Stem Cell-Associated Gene Expression Signature Allows Risk Stratification in Pediatric Acute Myeloid Leukemia. Leukemia.

[183] Bill M., Nicolet D., Kohlschmidt J., Walker C.J., Mrózek K., Eisfeld A.-K., Papaioannou D., Rong-Mullins X., Brannan Z., Kolitz J.E. (2019). Mutations Associated with a 17-Gene Leukemia Stem Cell Score and Its Prognostic Relevance in the Context of the European LeukemiaNet Classification for Acute Myeloid Leukemia. Haematologica.

[184] Vasseur L., Fenwarth L., Lambert J., de Botton S., Figeac M., Villenet C., Heiblig M., Dumas P., Récher C., Berthon C. (2023). LSC17 Score Complements Genetics and Measurable Residual Disease in Acute Myeloid Leukemia: An ALFA Study. Blood Adv..

[185] Legrand O., Simonin G., Perrot J.Y., Zittoun R., Marie J.P. (1998). Pgp and MRP Activities Using Calcein-AM Are Prognostic Factors in Adult Acute Myeloid Leukemia Patients. Blood.

[186] Laupeze B., Amiot L., Drenou B., Bernard M., Branger B., Grosset J.M., Lamy T., Fauchet R., Fardel O. (2002). High Multidrug Resistance Protein Activity in Acute Myeloid Leukaemias Is Associated with Poor Response to Chemotherapy and Reduced Patient Survival. Br. J. Haematol..

[187] Ong Y.L., McMullin M.F., Bailie K.E., Lappin T.R., Jones F.G., Irvine A.E. (2000). High Bax Expression Is a Good Prognostic Indicator in Acute Myeloid Leukaemia. Br. J. Haematol..

[188] Del Poeta G., Venditti A., Del Principe M.I., Maurillo L., Buccisano F., Tamburini A., Cox M.C., Franchi A., Bruno A., Mazzone C. (2003). Amount of Spontaneous Apoptosis Detected by Bax/Bcl-2 Ratio Predicts Outcome in Acute Myeloid Leukemia (AML). Blood.

[189] Venditti A., Poeta G.D., Maurillo L., Buccisano F., Principe M.D., Mazzone C., Tamburini A., Cox C., Panetta P., Neri B. (2004). Combined Analysis of Bcl-2 and MDR1 Proteins in 256 Cases of Acute Myeloid Leukemia. Haematologica.

[190] Carter B.Z., Qiu Y., Huang X., Diao L., Zhang N., Coombes K.R., Mak D.H., Konopleva M., Cortes J., Kantarjian H.M. (2012). Survivin Is Highly Expressed in CD34+38− Leukemic Stem/Progenitor Cells and Predicts Poor Clinical Outcomes in AML. Blood.

[191] Kornblau S.M., Tibes R., Qiu Y.H., Chen W., Kantarjian H.M., Andreeff M., Coombes K.R., Mills G.B. (2009). Functional Proteomic Profiling of AML Predicts Response and Survival. Blood.

[192] Jayavelu A.K., Wolf S., Buettner F., Alexe G., Häupl B., Comoglio F., Schneider C., Doebele C., Fuhrmann D.C., Wagner S. (2022). The Proteogenomic Subtypes of Acute Myeloid Leukemia. Cancer Cell.

[193] Vo T.-T., Ryan J., Carrasco R., Neuberg D., Rossi D.J., Stone R.M., Deangelo D.J., Frattini M.G., Letai A. (2012). Relative Mitochondrial Priming of Myeloblasts and Normal HSCs Determines Chemotherapeutic Success in AML. Cell.

[194] Dal Bello R., Pacchiardi K., Chauvel C., Adès L., Braun T., Pasanisi J., Fournier E., Berthon C., Clappier E., Raffoux E. (2023). Relative Mitochondrial Priming Predicts Survival in Older AML Patients Treated Intensively. HemaSphere.

[195] Benard B.A., Leak L.B., Azizi A., Thomas D., Gentles A.J., Majeti R. (2021). Clonal Architecture Predicts Clinical Outcomes and Drug Sensitivity in Acute Myeloid Leukemia. Nat. Commun..

[196] Miles L.A., Bowman R.L., Merlinsky T.R., Csete I.S., Ooi A.T., Durruthy-Durruthy R., Bowman M., Famulare C., Patel M.A., Mendez P. (2020). Single-Cell Mutation Analysis of Clonal Evolution in Myeloid Malignancies. Nature.

[197] Duchmann M., Micol J.-B., Duployez N., Raffoux E., Thomas X., Marolleau J.-P., Braun T., Adès L., Chantepie S., Lemasle E. (2021). Prognostic Significance of Concurrent Gene Mutations in Intensively Treated Patients with IDH-Mutated AML: An ALFA Study. Blood.

[198] Bochtler T., Stölzel F., Heilig C.E., Kunz C., Mohr B., Jauch A., Janssen J.W.G., Kramer M., Benner A., Bornhäuser M. (2013). Clonal Heterogeneity as Detected by Metaphase Karyotyping Is an Indicator of Poor Prognosis in Acute Myeloid Leukemia. J. Clin. Oncol..

[199] Li S., Garrett-Bakelman F.E., Chung S.S., Sanders M.A., Hricik T., Rapaport F., Patel J., Dillon R., Vijay P., Brown A.L. (2016). Distinct Evolution and Dynamics of Epigenetic and Genetic Heterogeneity in Acute Myeloid Leukemia. Nat. Med..

[200] Cerrano M., Duchmann M., Kim R., Vasseur L., Hirsch P., Thomas X., Quentin S., Pasanisi J., Passet M., Rabian F. (2021). Clonal Dominance Is an Adverse Prognostic Factor in Acute Myeloid Leukemia Treated with Intensive Chemotherapy. Leukemia.

[201] Morita K., Wang F., Jahn K., Hu T., Tanaka T., Sasaki Y., Kuipers J., Loghavi S., Wang S.A., Yan Y. (2020). Clonal Evolution of Acute Myeloid Leukemia Revealed by High-Throughput Single-Cell Genomics. Nat. Commun..

[202] Duchmann M., Laplane L., Itzykson R. (2021). Clonal Architecture and Evolutionary Dynamics in Acute Myeloid Leukemias. Cancers.

[203] Rodriguez-Meira A., Buck G., Clark S.-A., Povinelli B.J., Alcolea V., Louka E., McGowan S., Hamblin A., Sousos N., Barkas N. (2019). Unravelling Intratumoral Heterogeneity through High-Sensitivity Single-Cell Mutational Analysis and Parallel RNA Sequencing. Mol. Cell..

[204] Duchmann M., Joudinaud R., Boudry A., Pasanisi J., Di Feo G., Kim R., Bucci M., Chauvel C., Chat L., Larcher L. (2022). Hematopoietic Differentiation at Single-Cell Resolution in NPM1-Mutated AML. Blood Cancer J..

[205] Cornelissen J.J., Blaise D. (2016). Hematopoietic Stem Cell Transplantation for Patients with AML in First Complete Remission. Blood.

[206] Pastore F., Dufour A., Benthaus T., Metzeler K.H., Maharry K.S., Schneider S., Ksienzyk B., Mellert G., Zellmeier E., Kakadia P.M. (2014). Combined Molecular and Clinical Prognostic Index for Relapse and Survival in Cytogenetically Normal Acute Myeloid Leukemia. J. Clin. Oncol..

[207] Peterlin P., Renneville A., Abdelali R.B., Nibourel O., Thomas X., Pautas C., de Botton S., Raffoux E., Cayuela J.-M., Boissel N. (2015). Impact of Additional Genetic Alterations on the Outcome of Patients with NPM1-Mutated Cytogenetically Normal Acute Myeloid Leukemia. Haematologica.

[208] Bezerra M.F., Lima A.S., Piqué-Borràs M.-R., Silveira D.R., Coelho-Silva J.L., Pereira-Martins D.A., Weinhäuser I., Franca-Neto P.L., Quek L., Corby A. (2020). Co-Occurrence of DNMT3A, NPM1, FLT3 Mutations Identifies a Subset of Acute Myeloid Leukemia with Adverse Prognosis. Blood.

[209] Hernández Sánchez A., Villaverde Ramiro A., Sträng E., Gastone C., Heckman C.A., Versluis J., Abáigar M., Sobas M.A., Azibeiro Melchor R., Benner A. (2022). Machine Learning Allows the Identification of New Co-Mutational Patterns with Prognostic Implications in NPM1 Mutated AML—Results of the European Harmony Alliance. Blood.

[210] Gerstung M., Papaemmanuil E., Martincorena I., Bullinger L., Gaidzik V.I., Paschka P., Heuser M., Thol F., Bolli N., Ganly P. (2017). Precision Oncology for Acute Myeloid Leukemia Using a Knowledge Bank Approach. Nat. Genet..

[211] Huet S., Paubelle E., Lours C., Grange B., Courtois L., Chabane K., Charlot C., Mosnier I., Simonet T., Hayette S. (2018). Validation of the Prognostic Value of the Knowledge Bank Approach to Determine AML Prognosis in Real Life. Blood.

[212] Bill M., Mrózek K., Giacopelli B., Kohlschmidt J., Nicolet D., Papaioannou D., Eisfeld A.-K., Kolitz J.E., Powell B.L., Carroll A.J. (2021). Precision Oncology in AML: Validation of the Prognostic Value of the Knowledge Bank Approach and Suggestions for Improvement. J. Hematol. Oncol..

[213] Fenwarth L., Thomas X., de Botton S., Duployez N., Bourhis J.-H., Lesieur A., Fortin G., Meslin P.-A., Yakoub-Agha I., Sujobert P. (2021). A Personalized Approach to Guide Allogeneic Stem Cell Transplantation in Younger Adults with Acute Myeloid Leukemia. Blood.

[214] Itzykson R., Fournier E., Berthon C., Röllig C., Braun T., Marceau-Renaut A., Pautas C., Nibourel O., Lemasle E., Micol J.-B. (2021). Genetic Identification of Patients with AML Older than 60 Years Achieving Long-Term Survival with Intensive Chemotherapy. Blood.

[215] Venugopal S., Borthakur G., Daver N., DiNardo C.D., Pemmaraju N., Short N.J., Abbas H.A., Garcia-Manero G., Konopleva M., Ravandi F. (2022). Validation of ALFA 1200 Score in Patients with AML >60 Years Treated with Double Nucleoside-Based Low-Intensity Therapy. Blood Adv..

[216] Cerrano M., Itzykson R. (2019). New Treatment Options for Acute Myeloid Leukemia in 2019. Curr. Oncol. Rep..

[217] Calleja A., Loschi M., Bailly L., Morisot A., Marceau A., Mannone L., Robert G., Auberger P., Preudhomme C., Raynaud S. (2023). Real-Life Challenges Using Personalized Prognostic Scoring Systems in Acute Myeloid Leukemia. Cancer Med..

[218] Herrmann L., Bischof L., Backhaus D., Brauer D., Franke G.-N., Vucinic V., Platzbecker U., Schwind S., Jentzsch M. (2022). Outcome Prediction by the Knowledge Bank Approach in Acute Myeloid Leukemia Patients Undergoing Allogeneic Stem Cell Transplantation. Am. J. Hematol..

[219] Letai A., Bhola P., Welm A.L. (2022). Functional Precision Oncology: Testing Tumors with Drugs to Identify Vulnerabilities and Novel Combinations. Cancer Cell.

[220] Tyner J.W., Tognon C.E., Bottomly D., Wilmot B., Kurtz S.E., Savage S.L., Long N., Schultz A.R., Traer E., Abel M. (2018). Functional Genomic Landscape of Acute Myeloid Leukaemia. Nature.

[221] Dal Bello R., Pasanisi J., Joudinaud R., Duchmann M., Pardieu B., Ayaka P., Di Feo G., Sodaro G., Chauvel C., Kim R. (2022). A Multiparametric Niche-like Drug Screening Platform in Acute Myeloid Leukemia. Blood Cancer J..

[222] Walter R.B., Estey E.H. (2020). Selection of Initial Therapy for Newly-Diagnosed Adult Acute Myeloid Leukemia: Limitations of Predictive Models. Blood Rev..

